# Entropy Pair Functional Theory: Direct Entropy Evaluation Spanning Phase Transitions

**DOI:** 10.3390/e23020234

**Published:** 2021-02-17

**Authors:** Donald M. Nicholson, C. Y. Gao, Marshall T. McDonnell, Clifton C. Sluss, David J. Keffer

**Affiliations:** 1 Department of Physics and Astronomy, University of North Carolina, Asheville, NC 28803, USA; 2 Oak Ridge National Laboratory, Oak Ridge, TN 37831, USA; gaoy1@ornl.gov (C.Y.G.); mcdonnellmt@ornl.gov (M.T.M.); 3 Department of Materials Science and Engineering, University of Tennessee, Knoxville, Knoxville, TN 37996, USA; csluss@vols.utk.edu (C.C.S.); dkeffer@utk.edu (D.J.K.)

**Keywords:** entropy, free energy, entropy functional, pair correlation function, pair distribution function

## Abstract

We prove that, within the class of pair potential Hamiltonians, the excess entropy is a universal, temperature-independent functional of the density and pair correlation function. This result extends Henderson’s theorem, which states that the free energy is a temperature dependent functional of the density and pair correlation. The stationarity and concavity of the excess entropy functional are discussed and related to the Gibbs–Bugoliubov inequality and to the free energy. We apply the Kirkwood approximation, which is commonly used for fluids, to both fluids and solids. Approximate excess entropy functionals are developed and compared to results from thermodynamic integration. The pair functional approach gives the absolute entropy and free energy based on simulation output at a single temperature without thermodynamic integration. We argue that a functional of the type, which is strictly applicable to pair potentials, is also suitable for first principles calculation of free energies from Born–Oppenheimer molecular dynamics performed at a single temperature. This advancement has the potential to reduce the evaluation the free energy to a simple modification to any procedure that evaluates the energy and the pair correlation function.

## 1. Introduction

The Helmholtz free energy, A(N,V,T), is central to the description of stable and metastable equilibrium (number of atoms, *N*, volume, *V*, and temperature, *T*). It is also the starting point for the treatment of relaxation toward equilibrium and the responses of systems near equilibrium to perturbations. Furthermore, local representations of A(N,V,T) are fundamental to spatial and temporal coarse graining of materials dynamics. We propose a functional approach to A(N,V,T) that will be useful for a wide range of materials, chemistry, and molecular biology problems.

**Figure 1:**
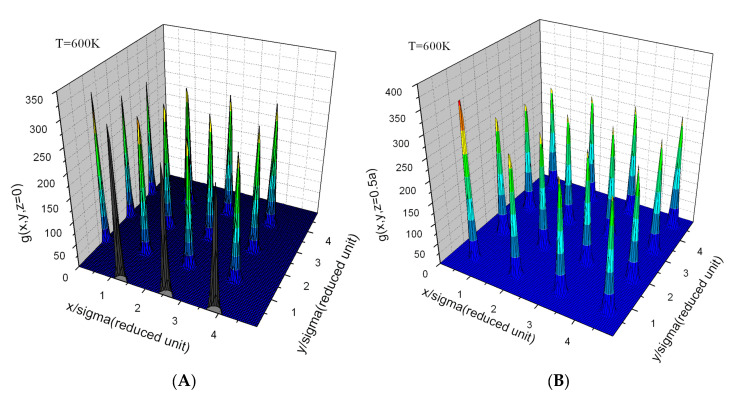
The pair correlation function, $\bar{g}\left(r\right)$, as defined by Equation (1) for BCC iron at 600 K as simulated with the Johnson potential [22]: (**A**) on a plane that passes through an atom at the origin and the four second nearest neighbors and (**B**) on a plane that passes through the four nearest neighbors of the atom at the origin. Sigma = 2.2143Å is the Sigma associated with the Lennard–Jones potential that very nearly matches the Johnson potential.

**Figure 2:**
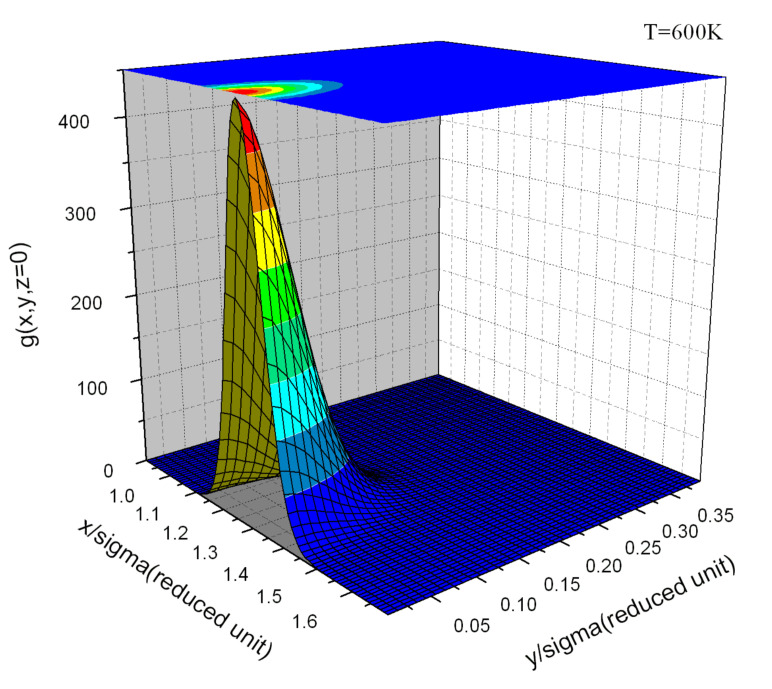
The second nearest neighbor peak (the peak closest to the origin in Figure 1A) of the pair correlation function $\bar{g}\left(r\right)$ at 600 K.

**Figure 3:**
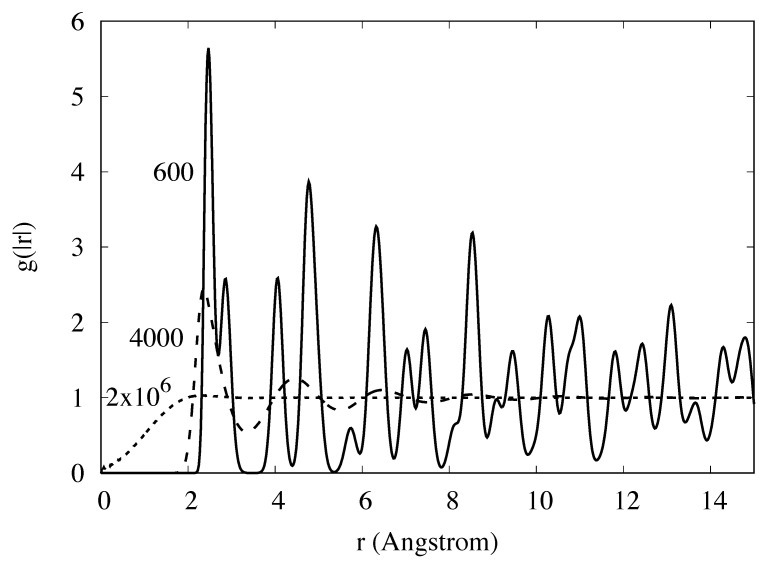
The pair correlation function $g_{s}\left(|r|\right)$ for BCC iron as simulated with the Johnson potential at 600, 4000, and 200,000 K.

The evaluation of A(N,V,T) is usually achieved by thermodynamic integration over temperature from the ideal gas or the harmonic crystal, or by integration over a parameter, λ, that continuously transforms one Hamiltonian with known free energy, $A_{\lambda =0}$, into the Hamiltonian of interest with free energy, $A_{\lambda =1}$ [1]. Methods for determining the free energy, such as thermodynamic integration, can be cumbersome [2], particularly for first principles Hamiltonians [3,4,5,6,7,8,9,10,11,12] and for experiments. In many studies, where knowledge of the free energy would be valuable, it is not calculated because of the complexity or computational expense involved in evaluation of the entropy. Alternative approaches should be explored. One approach employed for the treatment of fluids, referred to as “direct evaluation of the entropy” [13,14,15,16], is based on expansions of the probability density to increasing orders in the correlation functions and powers of the density, in which a limited set of terms is retained. Stopping at the pair level, the pair correlation functions (see typical pair correlation functions in Figure 1, Figure 2 and Figure 3) from simulations give an easily evaluated first approximation of the fluid entropy; if greater accuracy is sought, triplet correlations can be calculated and the triplet terms that contribute to the entropy can be evaluated [16,17]. Similarly, in the study of substitutional alloys, a cluster expansion of the configurational entropy is often evaluated by the cluster variation method (CVM) [18] in a process that includes increasingly higher levels of correlation to achieve the desired accuracy.

Here, we take a different approach. We prove the existence an excess entropy functional of the pair correlation, a universal functional that is valid for all systems. Our work falls within a long tradition of universal functional approaches (see Appendix A). Functional methods generally exploit stationary properties (see Appendix B).

Systems in equilibrium are characterized by time independent correlation functions as defined by Hansen and McDonald [19,20], i.e., the density, ρ(r), the pair correlation function (PCF), $g(r_{1},r_{2})$, and higher order correlation functions, $g^{\left(n\right)}\left(r_{1},r_{2}\cdots r_{n}\right)$. The correlation functions are defined in terms of the probability density, $P_{r}\left(r_{1}...r_{N}\right)$. $P_{r}\left(r_{1}...r_{N}\right)dr_{1}...dr_{N}$ is the probability that particle-*n* is in $dr_{n}$ for all n=1,n=2,...,n=N. In Equation (1) the atom coordinates appear in an ordered list from 1 to *N*. *P* is symmetric under interchange of $r_{1}...r_{N}$; however, it is useful to assign specific ordering (see Appendix C) to the atomic positions. The correlation functions are:

(1) $$g^{\left(n\right)}\left(r_{1}...r_{n}\right)=\frac{N!}{\Pi_{i=1}^{n}\rho_{1}\left(r_{i}\right)\left(N-n\right)!}\int dr_{n+1}...dr_{N}P_{r}\left(r_{1}...r_{N}\right),$$

where

(2) $$\rho_{1}\left(r_{1}\right)=N\int dr_{2}...dr_{N}P_{r}\left(r_{1}...r_{N}\right)=<\sum_{j=1,N}\delta \left(r_{1}-r_{j}\right)>.$$

For homogeneous systems $\rho_{1}=\rho =N/V$. When n=2 Equation (2) the pair correlation function is obtained; it is closely related to scattering experiments on liquids, glasses, and crystals [21].

According to generalizations of Henderson’s theorem [23,24], systems in equilibrium at *T* with interaction potentials of order *n* are completely characterized by the correlation functions up to $g^{\left(n\right)}$. This follows from the fact that the set of n-body interactions $\bar{V}=\left\{v^{\left(1\right)},v^{\left(2\right)},\cdots v^{\left(n\right)}\right\}$ are determined uniquely by the set of correlation functions $g^{\left(1\right)},g^{\left(2\right)}\cdots g^{\left(n\right)}$. If the pair and all higher order interactions are specified and fixed, e.g., $v^{\left(2\right)}=1/r$ and $v^{\left(n\right)}=0$ for n>2, then the density, $g^{\left(1\right)}\left(r\right)\equiv \rho \left(r\right)/\rho$ determines the point potential, $v^{\left(1\right)}\left(r\right)$, and hence all other properties. The implication that A(N,VT) is a functional of the density is the basis of classical density functional theory [25]. The reader familiar with classical density functional theory or with electron density functional theory will be comfortable with this statement and may feel that because in nature the interactions are fixed there is no need to go beyond functionals of the density. However, expressions for the functional dependence of A(N,V,T) can be improved and the process for their development for different pair interactions can be streamlined. Currently, a new functional of the density must be introduced each time a system with a new pair potential is explored [26,27,28,29]. Functionals of pair correlations may be useful for these objectives.

Furthermore, functionals of the pair correlation may be useful in a wider context. For example, consider that the pair correlation can be defined and measured as a function of time for non-equilibrium ensembles that may have dynamics whose governing equations can be derived from functional derivatives of the excess entropy, $S_{x}$, and A(N,V,T) [30]. The formalism presented here may thus have a wider range of application, beyond equilibrium. In this context the correlation functions provide a set of coordinates for coarse-grained dynamics [31] that may extend the time and spatial extent of simulation. Rather than elaborate on our vision for the use of this functional approach we simply make the surprising observation that the determination of the excess entropy requires only one quantity, the pair correlation function. For example, if the pair correlation function is known the excess entropy can be determined without knowledge of the temperature. This observation is sufficient to motivate our discussion. We also propose preliminary approximations to the universal excess-entropy functional of the density and pair correlation that require no knowledge of the interaction potentials or the temperature. We add the caveat that for the functional to be strictly applicable the Hamiltonian must be of pair potential form. However, we argue that higher order interactions are often small enough that they can be ignored because they affect the entropy only in second order (Appendix D). In this manuscript *N*, *V*, and *T* are assumed fixed and will be dropped from arguments henceforth.

The paper proceeds as follows. Section 2 describes properties of a variational form of the free energy. This form is combined with a constrained search to achieve a free energy functional of the density and pair correlation function. In Section 3 that part of the free energy that is independent of potentials and has an explicit linear dependence on T is identified with the excess-entropy, pair-density functional, $S_{x}\left[g\right]$; its properties are discussed for the homogeneous pair potential case. In Section 4 approximations to $S_{x}\left[g\right]$ that are suitable for all phases are developed. In developing these approximations we begin with the conventional Kirkwood approximation for fluid probability densities [1]. We then propose a modified Kirkwood approximation that is suitable for crystals and fluids. For the crystal a connection is made between the modified Kirkwood approximation, conditional pair probabilities, and harmonic approximations. Concluding remarks in Section 5 are followed by appendices. Appendix A gives a brief survey of universal functionals that will remind the reader of the similarities and differences between several uses of this type of construct. The relationship between the variation of the free energy and the pair interaction is discussed in Appendix B. Appendix C covers organizing the list of atomic coordinates. Appendix D shows that the effect on the excess entropy of higher order interactions is second order in their strength. Appendix E describes simulation that provide PCFs and excess entropy that are compared to the output of excess entropy functionals. The results are for the Johnson potential [22]; they include the construction of an essentially exact target excess entropy as a function of temperature. Finally, the variance of the crystalline separation vectors within the classical Debye model is given in Appendix F.

## 2. Free Energy Properties

*A* is usually expressed in terms of the partition function. In order to arrive at a form that is stationary with respect to variations of the probability density about its equilibrium value [25,32], we rewrite *A* in terms of the Hamiltonian and the probability density, $P(p_{N},r_{N})$, where $r_{N}$ and $p_{N}$ are the *N* positions and momenta respectively [33].

(3) $$A_{H}\left[P\right]=Tr\left\{P\left(H+k_{B}T\ln\left(P\right)\right)+k_{B}T\ln\left(N!h^{3N}\right)\right\}$$

The stationarity of this functional with respect to perturbation of the probability density, δP, about the equilibrium probability density, $P_{0}^{H}$, for Hamiltonian, *H*, follows from

(4) $$\delta A_{H}=Tr{\left\{\delta P\left(H+k_{B}T\ln\left(P\right)\right\}+Tr\left\{\delta Pk_{B}T\right\}\right|}_{P=P_{0}^{H}}=0,$$

where Tr{δP}=0, $P_{0}^{H}=\exp\left(-\frac{H}{k_{B}T}\right)/Z_{H}$, and $Z_{H}=Tr\left\{\exp\left(-\frac{H}{k_{B}T}\right)\right\}$ have been used to obtain the second identity. It is easily verified that substituting $P_{0}^{H}$ into Equation (3) gives the usual expression for free energy in terms of the partition function, $Q_{H}=\frac{1}{N!h^{3N}}Z_{H}$:

(5) $$A_{H}^{0}=A_{H}\left[P_{0}^{H}\right]=-k_{B}T\ln\left(Q_{H}\right)$$

When evaluating $A_{H}$ at a probability that differs from $P_{0}^{H}$ by an amount ΔP that preserves the normalization of $P_{0}$, i.e., Tr{ΔP}=0, we observed:

(6) $$\begin{array}{ccc} A_{H}\left[P_{0}^{H}+\Delta P\right] & = & Tr\{\left(P_{0}^{H}+\Delta P\right)\left(H+k_{B}T\ln P_{0}^{H}\right)\}\\ & + & k_{B}T\left(\ln\left(N!h^{3N}\right)+Tr\{\left(P_{0}^{H}+\Delta P\right)\left(\ln\left(P_{0}^{H}+\Delta P\right)-\ln\left(P_{0}^{H}\right)\right\}\right) \end{array}$$

By the Gibbs inequality, the second trace in Equation (6) is positive; therefore, *A* is minimized by $P_{0}^{H}$.

(7) $$\begin{array}{ccc} A_{H}\left[P_{0}^{H}+\Delta P\right] & \geq & A_{H}\left[P_{0}^{H}\right] \end{array}$$

To summarize, evaluating the free energy functional, Equation (3), at a probability that differs from the exact probability, $P_{0}^{H}$, always increases the result.

From Equation (7) we can derive upper and lower bounds for the free energy associated with Hamiltonian, *H*, that are based on the free energies of approximate Hamiltonians, $H_{a}$ that have know free energies (possibly analytic free energies). The Gibbs–Bogoliubov inequality [34], provides an upper bound which relates the change in free energy to the difference between the Hamiltonian of interest, $H=H_{a}+\Delta \bar{V}$ and the approximate Hamiltonian, $H_{a}$. This follows immediately from Equation (7) by setting $H=H_{a}+\Delta \bar{V}$ and $\Delta P=P_{0}^{H_{a}}-P_{0}^{H}$:

(8) $$\begin{array}{ccc} A_{H_{a}+\Delta \bar{V}}\left[P_{0}^{H}+\Delta P\right] & \geq & A_{H}\left[P_{0}^{H}\right]\\ A_{H_{a}}\left[P_{0}^{H_{a}}\right]+Tr\left\{\Delta \bar{V}P_{0}^{H_{a}}\right\} & \geq & A_{H}\left[P_{0}^{H}\right] \end{array}$$

A lower bound may be found by interchanging the perturbed and unperturbed quantities in Equation (8) [35]; i.e., $H_{a}⇌H=H_{a}+\Delta \bar{V}$, $P_{0}^{H_{a}}⇌P_{0}^{H}$, and ${\bar{V}}_{a}-\bar{V}⇌\bar{V}-{\bar{V}}_{a}$ or equivalently $-\Delta \bar{V}⇌+\Delta \bar{V}$

(9) $$\begin{array}{ccc} A_{H}\left[P_{0}^{H}\right]-Tr\left\{\Delta \bar{V}P_{0}^{H}\right\} & \geq & A_{H_{a}}\left[P_{0}^{H_{a}}\right]\\ A_{H_{a}}\left[P_{0}^{H_{a}}\right]+Tr\left\{\Delta \bar{V}P_{0}^{H}\right\} & \leq & A_{H}\left[P_{0}^{H}\right] \end{array}$$

An interesting situation emerges when all terms dependent on $\bar{V}$ are moved to the right-hand side:

(10) $$\begin{array}{ccc} A_{H_{a}}\left[P_{0}^{H_{a}}\right]-Tr\left\{{\bar{V}}_{a}P_{0}^{H}\right\} & \leq & A_{H}\left[P_{0}^{H}\right]-Tr\left\{\bar{V}P_{0}^{H}\right\}\\ S_{H}\left(P_{0}^{H}\right) & \leq & \;-\frac{1}{T}\left(A_{H_{a}}\left[P_{0}^{H_{a}}\right]-Tr\left\{P_{0}^{H}{\bar{V}}_{a}\right\}\right) \end{array}$$

If the trial Hamiltonian, $H_{a,\{c_{i}\}}$, is specified by adjustable interactions, ${\bar{V}}_{a,\{c_{i}\}}$, with parameters, $\left\{c_{i}\right\}$, e.g., ${\bar{V}}_{a,c}\left(r\right)=\frac{c}{r}$, then:

(11) $$\begin{array}{ccc} S_{H}\left(P_{0}^{H}\right) & \leq & \min_{\left\{c_{i}\right\}}-\frac{1}{T}\left(A_{H_{a,\{c_{i}\}}}\left[P_{0}^{H_{a,\{c_{i}\}}}\right]-Tr\left\{\left(P_{0}^{H}\right)\left({\bar{V}}_{a,\{c_{i}\}}\right)\right\}\right) \end{array}$$

Looking back at Equation (7), the minimum property of the free energy functional, we see that it also allows the following exact, explicit expression for the free energy that invokes two levels of minimization. First, for given momentum density, m(r), number density, ρ(r), and the pair correlation function, $g(r,r^{\prime })$, minimization is performed over all *P* that integrate to m(r),ρ(r), and $g(r,r^{\prime })$. Second, *A* is minimized over m(r),ρ(r), and $g(r,r^{\prime })$;

(12) $$A_{H}\left[m,\rho ,g\right]\equiv \min_{P\to m,\rho ,g}Tr\left\{P\left(H+k_{B}T\ln\left(P\right)+k_{B}T\ln\left(N!h^{3N}\right)\right)\right\};$$

(13) $$A_{H}=\min_{m,\rho ,g}A_{H}\left[m,\rho ,g\right].$$

Equation (12) summarizes the process of searching over all probability densities that give the specific *m*, ρ, and *g* and selecting the one that minimizes the trace. This is similar to the constrained search treatment of electron density functional theory [36]. Minimization over the momentum density (Equation (13)) can be easily performed because it is set by the temperature and can be removed from consideration because the probability density is separable, $P\left(p_{N},r_{N}\right)=P_{p}\left(p_{N}\right)P_{r}\left(r_{N}\right)=P_{p}\left(p_{N}\right)V^{-N}P_{r}\left(r_{N}\right)V^{N}$. Of all $P_{p}\left(p_{N}\right)$ that yield a specific *m* we choose $P_{p}\left(p_{N}\right)$ that gives the lowest *A* and further choose the $m=m_{min}$ that gives the lowest *A*. As a result, the free energy of the ideal gas, $A^{id}$, appears as a separate term in the total free energy.

AH=Tr{PpHp+PrHr+kBTPpln(PpV−N)+kBTPrln(PrVN)}+kBTln(N!h3N)(14)AH=Aid+Tr{PrHr+kBTPrln(PrVN)}(15)Aid=(−1+lnρΛ3)NkBT

where $\Lambda =\sqrt{\frac{2\pi \hbar^{2}}{mk_{B}T}}$ and the trace operation is understood to act on p or r according to context. Specializing to pair potential Hamiltonians, $\bar{V}=\left\{v^{\left(1\right)}\left(r\right),v^{\left(2\right)}\left(r,r^{\prime }\right)\right\}$:

(16) $$\begin{array}{ccc} A_{v}\left[\rho ,g\right]\equiv A\left[m_{min},\rho ,g\right]=A^{id} & + & \int drv^{\left(1\right)}\left(r\right)\rho \left(r\right)+\frac{1}{2}\int drdr^{\prime }\rho \left(r\right)g\left(r,r^{\prime }\right)v^{\left(2\right)}\left(r,r^{\prime }\right)\rho \left(r^{\prime }\right)\\ & + & T\max_{P_{r}\to \rho ,g}Tr\left\{k_{B}P_{r}\ln P_{r}V^{N}\right\} \end{array}$$

The “$v^{\left(1\right)}$ term” is the external potential energy; the “$v^{\left(2\right)}$ term” is the interaction energy. Subtracting these two potential energy terms from the excess free energy leaves the excess entropy term which has explicit dependence on *T* and implicit dependence through *g*. Dividing the “entropy term” by −T removes all explicit dependence on *T* and gives the excess entropy functional, $S_{x}^{\mathrm{exact}}\left[\rho ,g\right]$:

(17) $$S_{x}^{\mathrm{exact}}\left[\rho ,g\right]=-\max_{P_{r}\to \rho ,g}Tr\left\{k_{B}P_{r}\ln P_{r}V^{N}\right\}.$$

Specializing to $v^{\left(1\right)}=0$, fixed *N*, and fixed *V* (ρ=N/V) gives,

(18) $$S_{x}^{\mathrm{exact}}\left[g\right]=-\max_{P_{r}\to g}Tr\left\{k_{B}P_{r}\ln P_{r}V^{N}\right\}.$$

This is our main result. Equation (18) gives an exact but unusable expression for the excess entropy; maximizing over all $P_{r}$ is not feasible. Equation (18) does, however, prove that in any simulation (or experiment) the excess entropy depends on parameters such as temperature, particle mass, and pair potential only through their impact on *g*. $S_{x}\left[g\right]$ is a universal functional of the PCF; this simply means that it works without modification when applied to any pair potential simulation. In the sections below we address questions about the properties of the exact universal functional: (1) Can any properly normalized *g* be inserted into the functional? (2) What is the curvature of the functional? (3) How much does the value returned by the functional change when small changes to δg(r), are made in g(r)? (4) Is every *g* obtainable from a potential; is *g* “v representable”? Our development exactly parallels the development of electron density functional theory (see Appendix A). The next steps are to build upon theoretical and simulation results for the purpose of constructing useful approximations to the unusable exact functional and to compare the approximate values of the entropy to accurate values from thermodynamic integration of simulation results.

## 3. Excess Entropy Pair Density Functional

The subject of this paper, entropy in terms of correlation, is most clearly addressed by treating homogeneous pair potentials, $v^{\left(2\right)}\left(r_{1},r_{2}\right)=v\left(r=r_{2}-r_{1}\right)=v\left(r\right)$ of finite range in the homogeneous case, $v^{\left(1\right)}=0$. When $v^{\left(1\right)}=0$ it may be assumed, without loss of generality, that $\rho \left(r\right)=\rho =\frac{N}{V}$; crystallization takes place through spontaneous symmetry breaking in $g(r,r^{\prime })$. The free energy functional for a particular $v^{\left(2\right)}=v\left(r\right)$ separates into the potential energy, which has a simple explicit dependence on the potential, and the remaining kinetic and entropy terms; these remaining entropy terms form a universal functional of the pair correlation function. The form of the excess entropy is remarkable in having no explicit temperature dependence; knowledge of *g* and only *g* determines the excess entropy!

(19) $$\begin{array}{ccc} A_{v}^{x}\left[g\right]=A_{v}\left[g\right]-A^{id} & = & \frac{\rho }{2}\int dr_{1}drg\left(r_{1},r_{1}+r\right)v\left(r\right)-TS_{x}\left[g\right]\\ & = & \frac{N}{2}\int dr\bar{g}\left(r\right)v\left(r\right)-TS_{x}\left[g\right]\\ \bar{g}\left(r\right) & \equiv & \frac{1}{V}\int dr_{1}g\left(r_{1},r_{1}+r\right). \end{array}$$

We have introduced $\bar{g}$, an auxiliary functional of $g(r,r^{\prime })$ that will be used in development of entropy functionals. In most cases, v(r)=v(|r|)=v(r); therefore, it is often a useful simplification to use the functional, $g_{s}\left(|r|\right)\equiv \frac{1}{4\pi }\int^{\pi }sin\left(\theta \right)d\theta \int^{2\pi }d\phi \bar{g}\left(r,\theta ,\phi \right)$. In a homogeneous, isotropic fluid $g=\bar{g}=g_{s}$.

(20) $$A_{v}^{x}\left[g\right]=\frac{N}{2}\int drg_{s}\left(|r|\right)v\left(r\right)-TS_{x}\left[g\right]$$

$S_{x}$ is the exact, temperature independent excess-entropy functional of *g*:

(21) $$S_{x}\left[g\right]=\max_{P_{r}\to g}Tr\left\{-k_{B}P_{r}\ln P_{r}V^{N}\right\}.$$

The maximum excess entropy, Equation (18), a quantity with roots in statistical mechanics and information theory [37,38,39], with a constraint specified by *g*, is the excess entropy functional of *g*. This functional is defined over the domain of all *g* that can be derived from an *N* particle probability density, $P(r_{1},\cdots r_{N})$ by Equation (1)

(22) $$\begin{array}{c} \rho g\left(r_{1},r_{2}\right)\rho =N\left(N-1\right)\int dr_{3}...r_{N}P\left(r_{1}...r_{N}\right) \end{array}$$

or if the density is treated as inhomogeneous [40]:

(23) $$\begin{array}{ccc} \rho \left(r_{1}\right)g^{r}\left(r_{1},r_{2}\right)\rho \left(r_{2}\right) & = & N\left(N-1\right)\int dr_{3}...r_{N}P\left(r_{1}...r_{N}\right). \end{array}$$

The relationship between *g* and $g^{r}$ is:

(24) $$\begin{array}{ccc} g^{r}\left(r_{1},r_{2}\right) & = & \frac{g(r_{1},r_{2})}{g_{\infty }\left(r_{1},r_{2}\right)}\\ g_{\infty }\left(r_{1},r_{2}\right) & = & \frac{\rho \left(r_{1}\right)\rho \left(r_{2}\right)}{\rho^{2}}. \end{array}$$

We will often use simply *g*; if ambiguity is possible a specific subscript will be added, $g^{r}$ Equation (23) or $g_{H}$ for Equation (22).

What are the properties of the excess entropy functional? Are there only special *g* that form its domain or is the domain extensive and continuous? Can variations with respect to *g* be defined on the domain for the purpose of maximizing with respect to *g* or for finding the change in entropy associated with modification of *g*. Is the concavity determined over the domain so that if a maximum is found it is known to be the absolute maximum? Are all pair correlation functions related to at least one $P(r_{1}...r_{N})$ by Equation (24)?

For any $g_{1}$ and $g_{2}$ that are in the domain of $S_{x}$ then $\bar{g_{\lambda }}=\left(1-\lambda \right)g_{1}+\lambda g_{2}$ is in the domain. Furthermore, $S_{x}$ can be demonstrated to be concave, i.e., $S_{x}\left[\bar{g_{\lambda }}\right]\geq \left(1-\lambda \right)S_{x}\left[g_{1}\right]+\lambda S_{x}\left[g_{2}\right]$. This is shown by utilizing probability densities, $P_{1}$ and $P_{2}$, that are associated with $g_{1}$ and $g_{2}$ through the maximization process in Equation (18). We also introduce $\bar{P_{\lambda }}=\left(1-\lambda \right)P_{1}+\lambda P_{2}$, which importantly is not guaranteed to maximize the trace in Equation (18); therefore:

(25) $$S_{x}\left[\bar{g_{\lambda }}\right]\geq Tr\left\{-k_{B}\bar{P_{\lambda }}\ln\bar{P_{\lambda }}V^{N}\right\}.$$

Furthermore,

(26) $$\begin{array}{ccc} Tr\{-k_{B}\bar{P_{\lambda }}\ln\bar{P_{\lambda }}V^{N}\} & = & \left(1-\lambda \right)S_{x}\left(g_{1}\right)+\lambda S_{x}\left(g_{2}\right)\\ & + & k_{B}\left(\left(1-\lambda \right)Tr\left\{P_{1}\ln P_{1}-P_{1}\ln\bar{P_{\lambda }}\right\}+\lambda Tr\left\{P_{2}\ln P_{2}-P_{2}\ln\bar{P_{\lambda }}\right\}\right) \end{array}$$

The traces on the right hand side of Equation (26) are positive definite; therefore, $S_{x}$ is concave.

We can construct at least one proper probability density, the Kirkwood probability density [1], $P_{K}$. The Kirkwood product is symmetric and positive definite.

$$P_{K}\left(r_{N}\right)=\frac{\rho \left(r_{1}\right)\rho \left(r_{2}\right)...\;\rho \left(r_{\infty }\right)}{N!}\Pi_{i,j}^{N(N-1)/2}g\left(r_{i},r_{j}\right)\;$$

or

(27) $$P_{K}^{HT}\left(r_{N}\right)=\frac{\rho \left(r_{1}\right)\rho \left(r_{2}\right)...\;\rho \left(r_{\infty }\right)}{N!}\Pi_{i,j}^{N(N-1)/2}\left(g\left(r_{i},r_{j}\right)\left(1-\frac{1}{N}\right)\right)$$

The factors appearing in the first and second forms of the Kirkwood probability density in Equation (27) differ by terms or of order $\frac{1}{N}$. In the second form, $P_{K}^{HT}\left(r_{N}\right)$, the factor $\left(1-\frac{1}{N}\right)$ assures the correct High *T* limit, $\lim_{T\to \infty }P^{HT}\left(r_{N}\right)=\rho^{N}\frac{N!}{N^{N}}$ through first order in $\frac{1}{N}$ [13,41,42,43,44]. Integrating over ”$dr_{3}...dr_{N}$” gives g(1,2) because g(r)=1 for an amount of integration volume in $r_{3}...r_{N}$ that becomes infinite in the thermodynamic limit. The domain of the excess entropy functional is extensive; every *g* yields at at least one physically admissible probability density and therefore is within the domain of the entropy functional. Therefore, given any *g* a physically admissible probability can be constructed.

The first of Equations (27) is written below in a more informative way; it applies to both fluids and crystals:

(28) $$\begin{array}{ccc} N!P_{K}=g\left(0,1\right)\rho \left(1\right)g\left(1,2\right)\rho \left(2\right)g\left(2,3\right)\rho \left(3\right)g\left(3,4\right) & ... & g(N-2,N-1)\rho (N-1)\\ \;\;g(0,2)\;\;\;\;\;\;g(1,3)\;\;\;\;\;\;g(2,4) & ... & g(N-2,N-1)\\ \;\;\;\;\;g(0,3)\;\;\;\;\;\;g(1,4) & ... & g(N-2,N-1)\\ \;\;\;\;\;\;\;\;g(0,4) & ... & g(N-2,N-1). \end{array}$$

If the atoms are ordered along a chain as described in Appendix C, we can think of the first line of Equation (28) as representing a path from nearest neighbor to nearest neighbor; the second line goes from second neighbor to second neighbor (second neighbors along the chain but not necessarily in the structure). Let us look carefully at the entropy of the crystal. An exact expression for the probability density based on conditional pair probabilities [45] serves as a good starting point. $P^{\mathrm{crystal}}$ is given in terms of conditional probability densities, e.g., the probability that an atom is at $r_{2}$ given that an atom is at $r_{1}$ and with the condition that there is an atom at $r_{0}$, g(1,2|0)ρ(2):

(29) $$P^{\mathrm{crystal}}=\frac{1}{N!}g\left(0,1\right)\rho \left(1\right)g\left(1,2|0\right)\rho \left(2\right)g\left(2,3|0,1\right)\rho \left(3\right)g\left(3,4|0,1,2\right)\rho \left(4\right)...$$

Removing the conditions on each factor results in a non-symmetric approximation of the crystal probability (compare to Equation (28)):

(30) $$P_{\mathrm{non}}^{\mathrm{crystal}}=\frac{1}{N!}g\left(0,1\right)\rho \left(1\right)g\left(1,2\right)\rho \left(2\right)g\left(2,3\right)\rho \left(3\right)g\left(3,4\right)\rho \left(4\right)...$$

Comparing Equations (29) and (30) to the Kirkwood probability in Equation (28), we can identify the first line of Equation (28) with removal of conditions as in Equation (30). Each column in Equation (28) can be thought of as an approximation of a conditional probability in Equation (29); e.g.,

(31) $$\begin{array}{ccc} g(2,3|0,1) & \approx & g(2,3)g(1,3)g(0,2) \end{array}$$

## 4. Developing Approximations to the Entropy Functional

### 4.1. Kirkwood Entropy

The fact that the excess entropy is a universal functional allows us to propose approximations to *P* in terms of *g* and then to set parameters to give accurate entropies over many different pair potential simulations. In this paper we follow this approach; however, our dataset is very limited; we used simulation results for only the Johnson potential [22,46] from $T=10^{0}$ to $T=10^{7}$K. Simulations used to obtain g(r) and the target entropy are described in Appendix E.

Our choices for approximations are informed by three interrelated approaches:

1. The Kirkwood approximation for $P_{r}$ in terms of products of PCFs; Equation (28).
2. The exact expression for $P_{r}$ in terms of conditional probabilities (or *g*s); Equation (29).
3. The Morris–Ho method of entropy calculation in terms of simulated correlation [35].

Approximations to $P(r_{N})$ in terms of *g* can be directly inserted into Equation (18) to generate approximate functionals [47]. Substituting the Kirkwood probability density into Equation (18):

(32) $$s_{K}^{x}\left[g\right]=-1-\frac{1}{2}\{\int_{V}dr_{1}\int_{V}dr_{2}\left(P^{\left(2\right)}\left(r_{1}r_{2}\right)\ln g\left(r_{1},r_{2}\right)\right\},$$

Simplifying to a homogeneous fluid gives the Kirkwood entropy as proposed by Green and Wallace [1,13,14,15,41,42,43,44,48]:

(33) $$s_{K}^{x}\left[g\right]=-1+\lim_{R\to \infty }-\frac{1}{2}\left\{-1+\rho \int_{0}^{\infty }dr\bar{g}\left(r\right)\left(\ln\bar{g}\left(r\right)-\left(\bar{g}\left(r\right)-1\right)\right)\right\}.$$

Equation (33) has three problems:

1. Due to a normalization problem it does not go to the correct limit at high temperature [49].
2. It underestimates the entropy just above melting.
3. It grossly underestimates the entropy below melting; it gives −∞ for the crystal.

These shortcomings will undermine accuracy when applied to most systems. We understand the source of these shortcomings. The normalization problem at high temperature is addressed by the replacement g→gexp(ϕ[g]); ϕ is a parameterized functional of *g*. The cause of the Kirkwood overestimation of the entropy drop around melting is clear from Equation (27); in the fluid, where $\bar{g}\left(r\right)$ has broad peaks (Figure 3), multiple products are needed to maintain separation between atoms. For example, atoms that form a triangle could overlap if only two spherical *g*s were included in the product. However, in the low-temperature-crystal $\bar{g}\left(r\right)$ approaches a sum of delta functions (Figure 1); in this limit Equation (27) should be replaced by an expression with fewer constraining factors. The N(N−1)/2 factors in the Kirkwood approximation (count the factors in Equation (27)) should be continuously reduced as the liquid cools toward crystallization, they should tend toward (N−1) factors. Raising *g* to a fractional power, $g\to g^{\frac{1}{\gamma [g]}}$, effectively reduces the number of factors of *g* appearing in $P_{r}$; γ[g] is a parameterized functional of *g*. The choice $\gamma =\frac{N}{2}$ is equivalent to reducing the number of factors of *g* to exactly N−1. Combining the effects of ϕ and γ gives $g\to g^{\frac{1}{\gamma [g]}}e^{\phi [g]}$ Notice that in the thermodynamic limit it is immaterial whether the replacement $g\to g^{\frac{1}{\gamma [g]}}e^{\phi [g]}$ is applied to all *g* occurring in Equation (28) or if those on the first row are excluded from the substitution. Keep in mind that γ[g] and ϕ[g] are independent of r and *T*; however, they can depend on whether *g* has a form associated with a particular underlying crystal or fluid.

### 4.2. Modified Kirkwood Entropy Applied to Fluid Pair Correlation Functions

For the fluid, where ρ=N/V, the replacement, $g\to g^{\frac{1}{\gamma [g]}}\exp\phi [g]$, gives:

(34) $$\begin{array}{ccc} P_{K}^{M}=\;\;\;\;\;\;\;\;\;\;\;\;\;\;\;\;\;\;\;\;\;\;\;\;\;\;\;\;\;\;\;\\ \frac{\rho^{N}}{N!}{(g\left(0,1\right)}^{1/\gamma [g(0,1)]}e^{\phi [g(0,1)]}g{\left(1,2\right)}^{1/\gamma [g(1,2)]}e^{\phi [g(1,2)]}g{\left(1,3\right)}^{1/\gamma [g(2,3)]}e^{\phi [g(2,3)]} & ... & \\ \;\;g{\left(0,2\right)}^{1/\gamma [g(0,2)]}e^{\phi [g(0,2)]}g{\left(1,3\right)}^{1/\gamma [g(1,3)]}e^{\phi [g(1,3)]} & ... & \\ \;\;\;\;\;g{\left(0,3\right)}^{1/\gamma [g(0,3)]}e^{\phi [g(0,3)]} & ... & ) \end{array}$$

Using the modified Kirkwood probability density, $P_{K}^{M}$, to approximate the maximizing $P_{r}$ in the argument of the ln in Equation (18) gives an entropy functional that we refer to as the modified Kirkwood entropy, ${\tilde{s}}_{K}^{x}\left[g\right]$.

Specializing to a homogeneous phase and collapsing the effect of ϕ into a term, $\frac{1}{2}\tilde{\phi }$ gives:

(35) $${\tilde{s}}_{K}^{x}\left[g\right]=\frac{1}{2}\tilde{\phi }\left[g\right]-1+\lim_{R\to \infty }-\frac{1}{2}\left\{-1+\frac{\rho }{\gamma }\int_{0}^{R}dr\left(\bar{g}\left(r\right)\ln g\left(r\right)-\bar{g}\left(r\right)+1\right)\right\}$$

The term $\frac{1}{2}\tilde{\phi }$ will be selected to give the correct result for the Johnson Potential when *g* is almost independent of r, as it is at extremely high temperature. The value of $\tilde{\phi }$ should approach one at extremely high temperature where $g_{s}\left(|r|\right)=\left(1-1/N\right)$. Let us follow the behavior of $g_{s}$ for large *r* as the temperature is increased. Starting at melting and extending to a high temperature we observe that $\lim_{r\to \infty }g_{s}\left(|r|\right)=1$, then at an extremely high temperature, $T_{t}$, the large *r* value of $g_{s}$ undergoes a transition to, $\lim_{r\to \infty }g_{s}\left(|r|\right)=\left(1-1/N\right)$ (this limit is easily reached because we use a potential that is finite at r=0 [46]). Up to $T_{t}$ the excluded atom ($\rho \int dr\bar{g}=N-1)$ is compensated within the first few neighbor shells. Above $T_{t}$ the excluded atom is spread over the whole volume. At infinite *T* the excluded atom is spread evenly over the total volume. We characterize $\tilde{\phi }$ by the amount of the excluded atom that is inside the sphere inscribed in the simulation box, $R_{\mathrm{in}}$. The fractions of the volume inside and outside the sphere are: $f_{in}=\frac{4\pi }{3V}R_{\mathrm{in}}^{3}$ and $f_{\mathrm{out}}=1-f_{\mathrm{in}}$. Define:

(36) $$Q\left[g\right]=\frac{1-N_{\mathrm{diff}}}{f_{\mathrm{out}}},$$

where $N_{\mathrm{diff}}\equiv Nf_{\mathrm{in}}-\rho \int_{0}^{R_{\mathrm{in}}}drg_{s}$. *Q* characterizes the large *r* behavior of *g*; it transitions from zero at low temperature (Q is zero for the crystal) to one in the extreme high temperature range. We selected a form for $\tilde{\phi }$ that is parameterized in terms of *Q* by $q_{1}$ and $q_{2}$:

(37) $$\tilde{\phi }\left[g\right]=Q+q_{1}Q\left(1-Q\right)+q_{2}Q^{2}\left(1-Q\right)$$

This form of $\tilde{\phi }\left[g\right]$ guarantees that $\tilde{\phi }=0$ for the crystal and gives the proper behavior for the entropy in the extreme high temperature limit.

If the functionals γ[g] and ϕ[g] are constructed on physical grounds, they could provide a reasonable starting point for a universal excess entropy functional, even though they are fitted to limited data. We define some simple functionals upon which we can build parameterized forms for γ[g]. Observing $\bar{g}\left(r\right)$, it is straight forward to determine whether it corresponds to a crystal or to a fluid; if it corresponds to a crystal the particular crystal is also easy to determine (see Figure 1). We define the functional, $I_{p}\left[g\right]$ to be BCC, FCC, HCP, etc., or fluid.

The Kirkwood entropy overestimates the gradual drop in entropy above melting and overestimates the drop in entropy at melting. Therefore, we need a *T*-independent functional of *g* that indicates the approach to crystallization so that we can use it to construct a universal excess entropy functional with the correct entropy reduction near melting. Consider the functional:

(38) $$\begin{array}{ccc} \kappa [g] & = & \frac{\rho }{4\pi }\int drG^{2}\left(r\right)\\ G(r) & = & 4\pi rh(r)\\ h(r) & = & g_{s}\left(|r|\right)-1 \end{array}$$

The functional κ[g] increases in the fluid as *T* is reduced and diverges at crystallization [50]. Note that κ provides a monitor of the level of long-range correlation and the approach to crystallization. It can be used to construct an entropy functional that changes as crystallization is approached. For the fluid we restrict the dependence of γ on *g* to be through κ, i.e., γ(κ[g]). When $I_{p}=\mathrm{fluid}$ we choose a form,$\gamma \left[g\right]=1+q_{0}\kappa^{4}$, parameterized by $q_{0}\left(\rho \right)$ [51].

(39) $$\begin{array}{ccc} \gamma [g] & = & 1+q_{0}\kappa {\left[\bar{g}\right]}^{4}\;\mathrm{fluid} \end{array}$$

### 4.3. Kirkwood Entropy Applied to Crystal Pair Correlation Functions

A functional described by Equation (35) is intended to apply to fluid and crystalline phases. As the excess entropy functional has no explicit dependence on *T* it should apply as a system changes phase from fluid to crystal. At crystallization, entropy drops abruptly; the entropy change is referred to as the entropy of fusion. For crystals the PCFs are peaked at separation vectors that are difference vectors of the lattice. For the Johnson potential, these peaks are well represented by spherical Gaussians (Figure 1 and Figure 2). The PCF, $g^{H}$, is characterized by Gaussians of width, $\lambda_{0i}$ at the separations equal to $R_{i}$ of the average lattice [52,53,54].

(40) $$G_{\lambda_{0i}}\left(R_{i},r\right)=\frac{\exp(-|\frac{r-R_{i}}{\lambda_{0i}}{|}^{2})}{\rho {\left(\pi \right)}^{3/2}\lambda_{0i}^{3}}$$

(41) $$g^{H}\left(r\right)=\sum_{i}^{R_{i}<R_{\mathrm{in}}}G_{\lambda_{0i}}\left(R_{i},r\right)$$

The Gaussian width converges to $\lambda_{\infty }$ as the separation vector approaches $R_{\infty }$:

(42) $$G_{\lambda_{\infty }}\left(R_{\infty },r\right)=\frac{\exp(-|\frac{r-R_{\infty }}{\lambda_{\infty }}{|}^{2})}{\rho {\left(\pi \right)}^{3/2}\lambda_{\infty }^{3}}$$

Displacements of atoms from their average positions are uncorrelated when the site separation is large, denoted by $R_{\infty }$. Therefore, $\lambda_{\infty }^{2}=2\lambda_{00}^{2}$ where $\lambda_{00}^{2}/2$ is the variance of the atomic displacement. The displacement of every atom about its lattice site is described by the Gaussian:

(43) $$G_{\lambda_{00}}\left(r\right)=\frac{\exp(-|\frac{r}{\lambda_{00}}{|}^{2})}{\rho {\left(\pi \right)}^{3/2}\lambda_{00}^{3}}$$

The density that appears in the Kirkwood formula when applied to crystals is specified at each site by $\lambda_{00}$; $\rho_{i}\left(r-R_{i}\right)=\rho G_{\lambda_{00}}\left(r-R_{i}\right)$.The entropy when $g^{r}=1$, in terms of $\lambda_{00}$ is:

(44) $$\begin{array}{ccc} s_{K}\left[g^{r}=1\right] & = & s^{id}-1+\frac{3}{2}+\frac{3}{2}\ln\frac{\lambda_{00}^{2}}{{\bar{\ell }}^{2}}\\ s_{K}^{x}\left[g^{r}=1\right] & = & -1+\frac{3}{2}+\frac{3}{2}\ln\frac{\lambda_{00}^{2}}{{\bar{\ell }}^{2}} \end{array}$$

where $\ell^{3}=V/N$ and $\bar{\ell }=\frac{\ell }{\sqrt{\pi }}$. In $s_{K}^{x}\left[1\right]$ it can be recognized that the term −1 represents the restriction to one atom per site, the term $\frac{3}{2}$ is essentially the equipartition energy divided by T, and $\frac{3}{2}\ln\frac{\lambda_{00}^{2}}{\bar{\ell }}$ is the reduction in entropy because each atom is restricted to its site to within a range characterized by $\lambda_{00}$. As λs are proportional to $\sqrt{T}$ at low temperature the g=1 entropy and the improvements discussed below are closely related to the equipartition specific heat, $C_{v}=\left(\mathrm{degrees}\;\mathrm{of}\;\mathrm{freedom}\right)/2$. In our situation, the equipartition contribution to the excess entropy is $\int \frac{3}{2T}+\mathrm{integration}\;\mathrm{constant}$ (in *d* dimensions, $\frac{3}{2}\to \frac{d}{2}$). The choice of a particular expression for the excess entropy in terms of λ’s affects mainly the integration constant in the equipartition excess entropy. Note, the integration constant is important; it determines the entropy of fusion. The g=1 entropy is a reasonable extrapolation of the liquid entropy to low temperature. It misses the drop in entropy at melting. This makes sense because the g=1 entropy does not include any reduction in entropy associated with correlation between atoms on different sites. The Kirkwood approximation can guide us. One approach is to consider only the factors of g(r) in the first row of Equation (28), where $g=\frac{G_{\lambda_{01}}}{G_{\lambda_{\infty }}}$. This row has N factors of *g*; thus, this approach achieves the mission assigned to γ in the discussion of fluids; it reduces the factors of *g* by $\frac{N}{2}$. The first row gives:

(45) $$s_{K}^{x}\left[G_{\lambda_{01}}\right]=\frac{3}{2}\ln\frac{\lambda_{01}^{2}}{\lambda_{\infty }^{2}}+\frac{3}{2}\left(1-\frac{\lambda_{01}^{2}}{\lambda_{\infty }^{2}}\right).$$

The $i^{\mathrm{th}}$ row will contribute $\frac{3}{2}\ln\frac{\lambda_{i}^{2}}{\lambda_{\infty }^{2}}+\frac{3}{2}\left(1-\frac{\lambda_{i}^{2}}{\lambda_{\infty }^{2}}\right)$ to the entropy [55]. If only the first row (first nearest neighbors) is included the result is equivalent to neglect of the conditions on the conditional probabilities. If more rows are added, then contributions are included from neighbors beyond first neighbors; the contributions of these more distant neighbors will usually be smaller because their correlation diminishes with distance.

(46) $$\begin{array}{c} s_{K}^{x}\left[g\left(r\right)\right]=s_{x}\left[1\right]+\frac{3}{2}\sum_{i}\ln\frac{\lambda_{i}^{2}}{\lambda_{\infty }^{2}}+\frac{3}{2}\left(1-\frac{\lambda_{i}^{2}}{\lambda_{\infty }^{2}}\right) \end{array}$$

Perhaps the simplest approach is use the spherically averaged PCFs. If the PCFs are treated as spherical in Equation (32), the Kirkwood entropy, $s_{x}^{K}\left[g_{s}\right]$, is given by:

(47) $$\begin{array}{c} s_{K}^{x}[g_{s}\left(|r|)\right]=s_{K}^{x}\left[1\right]+s_{K}^{x}[g_{s}^{H}\left(|r|)\right]-s_{K}^{x}[g_{s|\infty }^{H}(|r|)]. \end{array}$$

In all cases, if there is no correlation between motion on different sites the entropy reverts to the entropy of the $g^{r}=1$ entropy.

**Figure 4:**
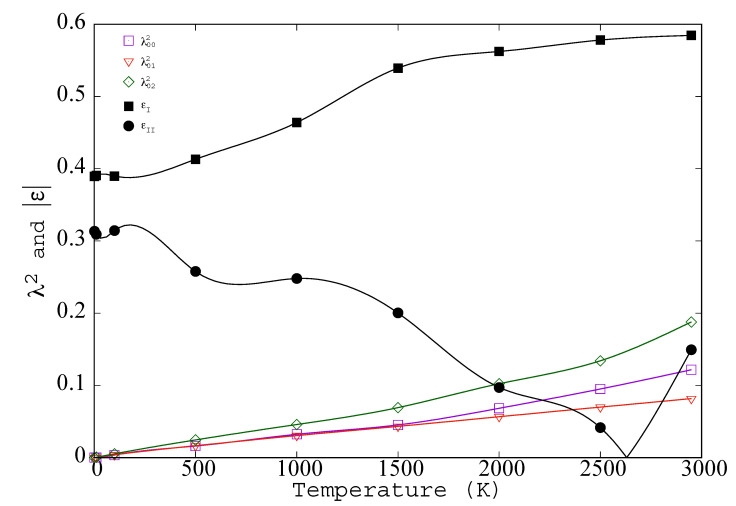
The simulated values of $\lambda_{00}^{2}$, $\lambda_{01}^{2}$, $\lambda_{02}^{2}$, and ϵ are plotted for BCC iron, as simulated with the Johnson potential (λs are in Angstroms and ϵ is dimensionless).

We have found that the values of $\lambda_{0i}$ can be determined by fitting $g_{s}\left(|r|\right)$ to the sum over $R_{i}$ of the integral over solid angles of $G_{\lambda_{i}}\left(R_{i},r\right)$; see Equation (41). The values of $\lambda_{0i}\left[g_{s}\right]$ obtained by fitting $g_{s}\left(|r|\right)$ are shown for $T<T_{m}$ in Figure 4. For crystals, the λs are just an alternative way of specifying g(r). At low temperature they are proportional to $\sqrt{T}$, as is to be expected in the regime where a harmonic model is valid. As sites move farther apart the peak widths, $\lambda_{0i}$, grow.

Recapping, the Kirkwood approximation of $P_{r}$ was applied to fluids and crystals. In fluids we proposed modification through ϕ and γ to correct the normalization of *g* and the multiplicity of *g*s that appear in $P_{r}$. Thus, for fluid PCFs we have discussed two possible approximations to the functional: (1) the original Kirkwood excess entropy and (2) the modified Kirkwood excess entropy. For Crystals, the excess entropy of the g=1 system is a reasonable extrapolation of the modified Kirkwood result for fluid PCFs. The crystal does not have a normalization problem and the number of factors of *g* is not a problem for the g=1 entropy. We explored several ways to include inter site correlation via *g*; the different treatments are specified by the different arguments for the functional $s_{K}^{x}\left[g\right]$: (I) $s_{K}^{x}\left[1\right]$ is the g=1 entropy, (II) $s_{K}^{x}\left[G_{\lambda_{1}}\left(R_{1},r\right)\right]$ indicates that the sum in Equation (46) includes only terms from the first row, (III) $s_{K}^{x}\left[g\left(r\right)\right]$ indicates the sum is over all neighbors of contributions from the full vector correlation, g(r), and (IV) $s_{K}^{x}[g_{s}\left(|r|)\right]$ indicates that the correlation is approximated through the spherical average of *g*. For crystals, Expressions I and II represent limited summations of the terms contributing to the Kirkwood entropy. The full Kirkwood entropy is expressed in III; it is the most complete expression of the Kirkwood approximation. Expression IIII is a spherical approximation of the full Kirkwood expression.

To emphasize that a single functional spans phase transitions by acting on any properly normalized functions, g(r) we write:

(48) $${\tilde{s}}_{K}^{x}\left[g\right]=\left\{\begin{array}{cc} \frac{1}{2}\tilde{\phi }\left[g\right]-1+\lim_{N\to \infty }-\frac{1}{2}\left(-1+\rho \int_{\Omega }dr\left(g_{s}\ln g_{s}-g_{s}+1\right)\right){\left(1+q_{0}\kappa^{4}\right)}^{-1}\; & \mathrm{fluid};\\ -1+\frac{3}{2}+\frac{3}{2}\ln\frac{\lambda_{00}^{2}}{{\bar{\ell }}^{2}}+\frac{3}{2}\sum_{i}\ln\frac{\lambda_{i}^{2}}{\lambda_{\infty }^{2}}+\frac{3}{2}\left(1-\frac{\lambda_{i}^{2}}{\lambda_{\infty }^{2}}\right)\; & \mathrm{crystal}. \end{array}\right.$$

In Equation (48) any *g* that is found to be “fluid like” is inserted into the modified Kirkwood approximation for the fluid; any *g* that is “crystal like” is inserted into the full Kirkwood approximation for the crystal.

In the next section, alternative treatments of the crystal are discussed from the perspective of a harmonic crystal.

### 4.4. Connection to the Harmonic Crystal

For pair correlation functions that are very sharply peaked at crystal lattice vectors there should be a connection between Equations (44)–(47) and the entropy of harmonic solids. Equation (11) relates entropy to a that of an approximate interaction ${\bar{V}}_{a,\{c_{i}\}}\left(r_{1},...r_{N}\right)$ parameterized by $\left\{c_{i}\right\}$ and the actual probability density; could Equation (11) be a tool that allows such a connection to be built?

(49) $$\begin{array}{ccc} S[g] & \leq & \min_{\left\{c_{i}\right\}}-\frac{1}{T}\left(A_{{\bar{V}}_{a,\{c_{i}\}}}-\int dr_{N}P\left[g\right]{\bar{V}}_{a,\{c_{i}\}}\left(r_{N}\right)-<E_{k}>\right) \end{array}$$

We point out that in Equation (49) the entropy is a functional of *g* because the trial potential is determined by minimization for each choice of *g*. We follow Morris and Ho [35,56]: who proposed taking the trial interaction to be harmonic. For harmonic interactions the variational choices are the “spring constants,” $\left\{m\omega_{1}^{2},...m\omega_{N}^{2}\right\}$, and the coordinate choice, $\left\{y_{1},...y_{N}\right\}$, which is a set of 3N linearly independent coordinates that specify the atomic positions, $y_{k}={\mathrm{Map}}_{k}\left(r_{1},...r_{N}\right)$. In our monoatomic homogeneous system, using trial harmonic interactions, ${\bar{V}}_{\left\{\omega_{k},{\mathrm{Map}}_{k}\right\}}^{\mathrm{harmonic}}\left(r_{1},...r_{N}\right)=\sum_{k}^{3N}\frac{1}{2}m\omega_{k}^{2}y_{k}{\left(r_{1},...r_{N}\right)}^{2}$,^57^] gives a functional:

(50) $$\begin{array}{ccc} S_{\mathrm{harmonic}}\left[g\right] & = & \min_{\left\{\omega_{k},{\mathrm{Map}}_{k}\right\}}-\frac{1}{T}\left(A_{\left\{\omega_{k}y_{k}\right\}}^{\mathrm{harmonic}}-\int dr_{N}P\left[g\right]\sum_{k}^{3N}\frac{m\omega_{k}^{2}y_{k}{\left(r_{N}\right)}^{2}}{2}-<E_{k}>\right) \end{array}$$

The analytic free energy of harmonic systems is known and is independent of the choice of coordinates; let us work it out for our case. First consider the Einstein model, a quadratic point potential that attracts atoms to lattice sites, $y_{k}=x_{k}-R_{k}$. Note that such a quadratic potential increases without bound as an atom moves away from a lattice site; some ground rules need to be specified when sites are brought together to form a solid. We could allow one specific atom in the potential at each site; this would result in, 3N 1-d harmonic factors in the partition function, $\exp{\left(-\frac{H_{1d}}{k_{B}T}\right)}^{3N}$. Another choice is to let atoms be spread without prejudice as to the number of atoms on a given site; this leads to $N^{N}3N$ factors. Our choice is to disallow multiple occupation of sites; this is closest to the observed simulated behavior of g(r). Therefore, we obtain N!3N factors. Furthermore, we consider the potential to be zero at lattice sites and to extend to the Wigner-Seitz boundary where it joins the potentials centered at neighboring sites. We have mandated that sites be singly occupied, this can be thought of as the result of a repulsive potential acting between atoms that is strong enough to absolutely exclude two atoms from being at the same site. Therefore, the N atoms can be distributed among the sites in N! ways giving the N!3N harmonic factors appearing in the argument of ln below. If the exclusion is not present there would instead be $N^{N}3N$ factors. All $\omega_{k}$ will be chosen independent of *k*, $\omega_{k}=\omega$; this is consistent with all choices for $\left\{y_{k}\right\}$ being equivalent to within a translation.

(51) $$\begin{array}{ccc} -\frac{A_{\mathrm{harmonic}}}{k_{B}T} & = & -\frac{A^{\mathrm{id}}}{k_{B}T}\\ & + & \ln\int dr_{N}\exp\left(-\frac{\sum_{i,J}\frac{1}{2}m\omega^{2}{\left(r_{i}-R_{J}\right)}^{2}+\sum_{i,j}\;\mathrm{penalty}\;\;r_{i}\;\mathrm{near}\;r_{j}\;}{k_{B}T}\right)-k_{B}N\ln V\\ -\frac{A_{\mathrm{harmonic}}}{T} & = & -\frac{A^{\mathrm{id}}}{T}+3Nk_{B}\ln\sqrt{\frac{2\pi k_{B}T}{m\omega^{2}}}-k_{B}N+k_{B}N\ln\rho \end{array}$$

In the second equation we have assumed that the atoms are far enough apart that the repulsive interaction does not influence the free energy; this assumption will break down just before melting. In applying Equation (49) the kinetic energy has been grouped with the free energy of the ideal gas to form $S^{id}$:

(52) $$\begin{array}{ccc} S_{\mathrm{harmonic}}\left[g\right] & = & \left\{-\frac{A^{\mathrm{id}}}{T}+\frac{3}{2}Nk_{B}\right\}\\ & + & \min_{\left\{\omega ,{\mathrm{Map}}_{k}\right\}}3Nk_{B}\ln\sqrt{\frac{2\pi k_{B}T}{m\omega^{2}}}-k_{B}N+k_{B}N\ln\rho +\sum_{k}^{3N}\frac{1}{2T}m\omega^{2}<{y_{k}}^{2}>\\ S_{\mathrm{harmonic}}^{x}\left[g\right] & = & \min_{\left\{\omega ,{\mathrm{Map}}_{k}\right\}}3Nk_{B}\ln\sqrt{\frac{2\pi k_{B}T}{m\omega^{2}}}-k_{B}N+k_{B}N\ln\rho +\frac{3N}{2T}m\omega^{2}<{y_{k}}^{2}> \end{array}$$

We perform the minimization with respect to $\omega_{k}$ by setting $\frac{dS^{\mathrm{harmonic}}}{d\frac{1}{2T}m\omega_{k}^{2}}=0$.

(53) $$\begin{array}{ccc} s_{\mathrm{harmonic}}^{x}\left[g\right] & = & -1+\frac{3}{2}+\frac{3}{2}\ln\frac{2<{y_{k}}^{2}>}{{\bar{\ell }}^{2}} \end{array}$$

A completely general way to write a linear mapping to $y_{k}$ is $y_{k}=x_{k}-R_{k}-\sum_{j\neq k}a_{k,j}\left(x_{j}-R_{j}\right)$. This representation emphasizes our goal of having a local functional of *g*. We are not going to minimize with respect to $\left\{a_{i,j}\right\}$; instead we begin by exploring the two cases motivated by: (I) Kirkwood g=1 entropy of the crystal $s_{x}^{K}\left[1\right]$, and (II) which includes the entropy reduction from first neighbor correlation. For case I, we pick the classical Einstein model, $y_{k}^{I}=x_{k}-R_{k}$; each atom is harmonically attached to a lattice site. Case II is a chain with harmonic links, $y_{k}^{II}=x_{k}-R_{k}-\left(x_{k+1}-R_{k+1}\right)$, where k+1, is a nearest neighbor along the chain. Note that for both cases $<y_{k}>=0$. In case I $<{\left(y_{k}^{I}\right)}^{2}>=\frac{1}{2}\lambda_{00}^{2}=\frac{1}{4}\lambda_{\infty }^{2}$. In case II $<{\left(y_{k}^{II}\right)}^{2}>=\frac{1}{2}\lambda_{01}^{2}$.

Case I:

(54) $$\begin{array}{ccc} s_{I\;h}^{x}\left[g\right] & = & -1+\frac{3}{2}+\frac{3}{2}\ln\frac{\lambda_{00}^{2}}{{\bar{\ell }}^{2}} \end{array}$$

Case II:

(55) $$\begin{array}{ccc} s_{\mathrm{II}\;h}^{x}\left[g\right] & = & -1+\frac{3}{2}+\frac{3}{2}\ln\frac{\lambda_{01}^{2}}{{\bar{\ell }}^{2}} \end{array}$$

These results should look familiar; case I is the excess entropy when $g^{r}=1$, Equation (44), and case II is similar to the Kirkwood expression truncated after first nearest neighbor row, $s_{\mathrm{II}\;h}^{x}\left[g\right]=s_{K}^{x}\left[G_{\lambda_{01}}\left(R_{01},r\right)\right]-\frac{3}{2}\left(1-\ln2-\frac{\lambda_{01}^{2}}{2\lambda_{00}^{2}}\right)$, Equation (46). An important point is that the Morris–Ho approach gives an upper bound on the excess entropy. Therefore it is perfectly reasonable to specify the entropy functional as being assigned the lower of the values from these two approximations. Thus, as one might expect, the entropy is dominated by the most narrowly constrained correlation peaks.

In $s_{\mathrm{II}\;h}^{x}\left[g\right]$ and $s_{K}^{x}\left[G_{\lambda_{01}}\left(R_{1},r\right)\right]$ the entropy depends only on correlation between atoms that are nearest neighbors. What is the influence of other neighbors? In the conditional probability expression the more distant neighbors enter through the conditions. In the Morris–Ho treatment a natural way to include correlation with other neighbors is to consider $<y_{k}^{2}>$ to be the diagonal element of the correlation matrix, $C_{k,k^{\prime }}=<y_{k}y_{k^{\prime }}>$ (see [35]). If the correlation matrix is diagonal, i.e., $C\left(k,k^{\prime }\right)=<y_{k}^{2}>\delta_{k,k^{\prime }}$, or if it is approximated as diagonal, $C_{k,k^{\prime }}^{\mathrm{Diagonal}}=C_{k,k^{\prime }}\delta_{k,k^{\prime }}$ then $\sum_{k}^{3N}\ln\left(<y_{k}^{2}>\right)=\ln\left|C^{\mathrm{Diagonal}}\right|$. Other truncations that leave *C* symmetric can be used to evaluate ln|C|, e.g., block diagonal. These approximations merely correspond to making alternative choices for the set $\left\{y_{k}\right\}$. Truncating *C* to a tridiagonal matrix, $C_{k,k^{\prime }}^{\mathrm{Tridiagonal}}=C_{k,k^{\prime }}\left(\delta_{k,k^{\prime }}+\delta_{k,k^{\prime }+1}+\delta_{k,k^{\prime }-1}\right)$ incorporates additional coupling between displacements on different sites when applied to case I and between different bonds when applied to case II.

Is truncation of $C{˗}_{i,j}^{\alpha ,\beta }$ to a diagonal or tridiagonal matrix reasonable? In the tridiagonal matrix, how much smaller are the off-diagonal elements than the diagonal elements? The ratios are (see Figure 4):

(56) $$\begin{array}{ccc} \epsilon_{I} & \equiv & \frac{C_{0,1}^{\alpha ,\alpha }\left(\left\{x_{k}\right\}\right)}{C_{0,0}^{\alpha ,\alpha }\left(\left\{x_{k}\right\}\right)}=\frac{<x_{0}^{\alpha }x_{1}^{\alpha }>}{<x_{0}^{\alpha }x_{0}^{\alpha }>}=-\frac{\frac{\lambda_{01}^{2}\left[\bar{g}\right]}{4}-\frac{\lambda_{0,0}^{2}\left[\bar{g}\right]}{2}}{\frac{\lambda_{0,0}^{2}\left[\bar{g}\right]}{2}} \end{array}$$

(57) $$\begin{array}{ccc} \epsilon_{\mathrm{II}} & \equiv & \frac{C_{0,1}^{\alpha ,\alpha }\left(\left\{\Delta x_{k}\right\}\right)}{C_{0,0}^{\alpha ,\alpha }\left(\left\{\Delta x_{k}\right\}\right)}=\frac{<\Delta x_{0}^{\alpha }\Delta x_{1}^{\alpha }>}{<\Delta x_{0}^{\alpha }\Delta x_{0}^{\alpha }>}=\frac{\frac{\lambda_{0,2}^{2}\left[\bar{g}\right]}{4}-\frac{\lambda_{01}^{2}\left[\bar{g}\right]}{2}}{\frac{\lambda_{01}^{2}\left[\bar{g}\right]}{2}} \end{array}$$

For the low temperature Johnson potential both $|\epsilon_{I}|$ and $|\epsilon_{\mathrm{II}}|$ are about 0.4, consistent with the classical Debye value (see Appendix F). However $|\epsilon_{I}|$ climbs to 0.6 while $|\epsilon_{II}|$ dives, crossing zero a few hundred degrees before melting when $\frac{\lambda_{02}^{2}}{2}=\lambda_{01}^{2}$. This “zero” is likely to occur whenever ϵ>0 at T=0 because the first nearest neighbor peak will maintain its shape as melting approaches but the second nearest neighbor peak will lose its integrity. As a result of the very different behavior of $\epsilon_{I}$ and $\epsilon_{\mathrm{II}}$ the impact of off diagonal correlation is very different in the two cases.

In the homogeneous case where all sites are equivalent this tridiagonal correlation matrix becomes Toeplitz and has an analytic determinant [58]. Approximating the full correlation matrix by a Tridiagonal Toeplitz matrix, $C^{TT}$:

(58) $$\begin{array}{ccc} C_{i,j}^{TT} & = & C_{0,0}^{TT}\left\{\begin{array}{cc} 1 & i=j;\\ \epsilon \; & i=j+1,i=j-1\\ 0\; & \mathrm{otherwise} \end{array}\right. \end{array}$$

(59) $$\begin{array}{ccc} \psi & = & \left\{\begin{array}{cc} \cosh^{-1}\left(\frac{1}{2\epsilon }\right) & \epsilon <\frac{1}{2}\\ \; & \;\\ \;\;\cos^{-1}\left(\frac{1}{2\epsilon }\right)\; & \epsilon \geq \frac{1}{2} \end{array}\right. \end{array}$$

(60) $$\begin{array}{ccc} \left|C^{TT}\right| & = & {\left(C_{0,0}^{TT}\right)}^{3N}\epsilon^{3N}\left\{\begin{array}{cc} \frac{\sinh((3N+1)\psi )}{\sinh(\psi )} & \epsilon <\frac{1}{2}\\ \; & \;\\ \;\;\frac{\sin((3N+1)\psi )}{\sin(\psi )}\; & \epsilon \geq \frac{1}{2}. \end{array}\right. \end{array}$$

where:

(61) $$\begin{array}{c} \mathrm{Case}\;I\;\;C_{0,0}^{TT}=\frac{\lambda_{00}^{2}}{2} \end{array}$$

(62) $$\begin{array}{c} \mathrm{Case}\;\mathrm{II}\;\;C_{0,0}^{TT}=\frac{\lambda_{01}^{2}}{2}. \end{array}$$

Therefore, for cases I and II, in $lim_{N\to \infty }$, the off-diagonal coupling simply adds a term,

$ℜ\left[\frac{3}{2}\ln\left(1+\frac{1}{2}\left(\sqrt{1-{4|\epsilon |}^{2}}-1\right)\right)\right]$, to the entropy:

(63) $$\begin{array}{c} {\tilde{s}}_{I\;h-\mathrm{TT}}^{x}={\tilde{s}}_{K_{I\;h}}^{x}+\frac{3}{2}\ln\left(1+\frac{1}{2}\left(\sqrt{1-4|\epsilon_{I}{|}^{2}}-1\right)\right) \end{array}$$

$$\begin{array}{c} {\tilde{s}}_{\mathrm{II}\;h-\mathrm{TT}}^{x}={\tilde{s}}_{K_{\mathrm{II}\;h}}^{x}+\frac{3}{2}\ln\left(1+\frac{1}{2}\left(\sqrt{1-4|\epsilon_{\mathrm{II}}{|}^{2}}-1\right)\right) \end{array}$$

In case I, for the Johnson potential, the off-diagonal correlation ratio is large, $\epsilon \approx \frac{1}{2}$; it reduces the entropy significantly. Correlation gives an entropy of fusion in line with the target value and with Richard’s rule [59]. Note that, $\frac{3}{2}\ln\left(1+\frac{1}{2}\left(\sqrt{1-{4|\epsilon |}^{2}}-1\right)\right){|}_{\frac{1}{2}}\approx -1$. In case II, bond-correlation gives only a small reduction in the entropy; in fact, ${\tilde{s}}_{\mathrm{II}\;h}^{x}$ and ${\tilde{s}}_{\mathrm{II}\;h-\mathrm{TT}}^{x}$ are so close that in the discussion in the following section only ${\tilde{s}}_{\mathrm{II}\;h-\mathrm{TT}}^{x}$ will be shown. Equation (63) are attractive in two ways: first, they give an upper bound to the entropy and second, they are simple. Clearly, there is room to improve on these functionals; near melting, even though ${\tilde{s}}_{I\;h-\mathrm{TT}}^{x}$ is formally an upper bound, it is observed to be slightly lower than the target entropy. This indicates a problem; a possible explanation is that the assumption that every atom stay tightly bound to a specific site is violated near melting.

For distant neighbors, correlation ratios designated by ϵ will be small; their effect on the entropy should be roughly $\frac{3}{2}\ln\left(1+\frac{1}{2}\left(\sqrt{1-{4|\epsilon |}^{2}}-1\right)\right)\approx -\frac{3}{2}\epsilon^{2}$. This may suggests a form that can be used to incorporate the influence on entropy reduction from the weak correlation between more distant bonds. As the system can be specified by 3N coordinates there are three local coordinates available for each site. The coordinates having the smallest variances will dominate entropy reduction. Some possible choices are: the displacements from a lattice site $\left(x_{0},y_{0},z_{0}\right)$, the coordinates of a specific nearest neighbor separation $\left(x_{01},y_{01},z_{01}\right)$, or the distances to three different close neighbors, $\left(r_{1},r_{2},r_{3}\right)$. This is reminiscent of the equal partition theorem in which each of the 3N coordinates adds $1/2k_{B}$ to the specific heat. Here, the the 3N coordinates with the smallest fluctuations contribute $1/2\ln\frac{\lambda^{2}}{\bar{\ell }}$. Normally these low variance coordinates will involve close neighbors. The largest contributors (most negative) will be placed on the diagonal of the correlation matrix where each of the three variances will contribute terms like $\frac{1}{2}\ln\frac{\lambda^{2}}{\bar{\ell }}$. Weaker contributors will enter through terms like $\frac{3}{2}\ln\left(1+\frac{1}{2}\left(\sqrt{1-{4|\epsilon |}^{2}}-1\right)\right)$ that represent weak correlations that couple the more strongly correlating coordinates.

As the temperature of a crystal increases all variances grow. The variance $\lambda_{00}^{2}/2$, increases, as does the variance of the vector separations between neighbors, e.g., $\lambda_{01}^{2}/2$ and $\lambda_{0,2}^{2}/2$ (see Figure 4). At all temperatures the variance at large separation is exactly twice, $\lambda_{00}^{2}/2$. However, as the temperature increases the separation at which this factor of two is reached becomes shorter and shorter. Above melting $\lambda_{00}$ and $\lambda_{\infty }$ go to *∞*, while “$\lambda_{01}$” in the radial direction goes to a width indicated by the nearest neighbor peak of $\bar{g}\left(r\right)$ and in the two perpendicular directions it goes effectively to *∞* because neighboring atoms can be found anywhere in the spherical shell at |r| [60].

### 4.5. Comparison of Selected Functionals with the Target Entropy

**Figure 5:**
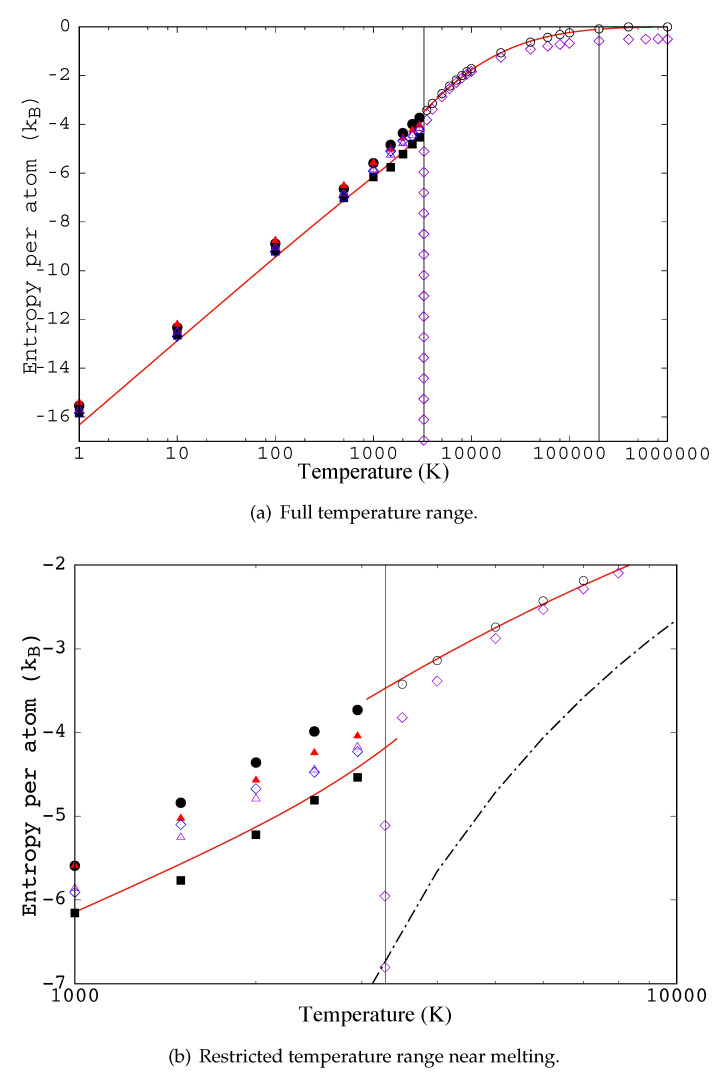
The target excess entropy shown in Figure A1 is reproduced here as a solid line with discontinuity at melting. The results of inserting $\bar{g}\left(r,r^{\prime }\right)$ from simulation with the Johnson potential at a set of temperatures into different functionals are shown. At each temperature, symbols represent the values of the various functionals (filled symbols indicate that they are formally upper bounds): (1) open diamonds show the Kirkwood entropy, $s_{K}^{x}$, for $T\geq T_{m}$ (see Equation (33)), and $s_{x}^{K}[g_{s}\left(|r|)\right]$ for $T<T_{m}$ (see Equation (47)); (2) the open circles show the modified Kirkwood entropy, ${\tilde{s}}_{K}^{x}$ (see Equation (35)); (3) filled circles show the g=1 entropy, $s_{x}^{K}\left[1\right]=s_{I\;h}^{x}$ (see Equation (44)); (4) filled triangles correspond to ${\tilde{s}}_{K_{\mathrm{II}\;h-\mathrm{TT}}}^{x}$ (see Equation (64)); (5) open triangles correspond to $s_{x}^{K}\left[g\left(r\right)\right]$ (see Equation (46)); and (6) filled squares are ${\tilde{s}}_{K_{I\;h-\mathrm{TT}}}^{x}$ (see Equation (63)). The vertical lines indicate $T_{m}$ and $T_{t}$. In Figure 6b the dot–dash line gives a classical density functional theory value for entropy based on the specific free energy functional proposed in Equation (40) of Lutsko and Lam [28] when evaluated for the Johnson potential. All results shown are for the same fixed volume and number.

Here we compare simulated values of the excess entropy for Jonhson-potential-iron to values from several approximate functionals. The functionals originate from several starting points as described above. We caution the reader not to discount approaches because of their poor performance in comparison to the simulated results. Further refinement of any of these approaches might mature into a very reliable functional. We have been reluctant to introduce fitting parameters. Agreement with simulated results can always be improved through more parameters. In our opinion it is best to wait until a broader array of approaches has been explored. Our only use of fitting parameters is for fluids approximated by the modified Kirkwood functional, ${\tilde{s}}_{K}^{x}$. It has three parameters, $q_{0}$, $q_{1}$, and $q_{2}$ [51], they were fit to the target excess entropy associated with the Johnson pair potential [22]. The target entropy was calculated by thermodynamic integration as described in Appendix E. The three parameters affect only the value of the functional for fluid pair correlation functions; furthermore, $q_{1}$ and $q_{2}$ affect only the fluid entropy at very high temperature. Agreement with the target in the fluid is not surprising; after all, we performed a fit. However, given the physical basis for the corrections, we anticipate that the values for $q_{0}$, $q_{1}$, and $q_{2}$ will perform reasonably for most systems. The modified Kirkwood entropy, ${\tilde{s}}_{K}^{x}$, is compared to the Kirkwood entropy, $s_{K}^{x}$, and the target in Figure 5.

**Figure 6:**
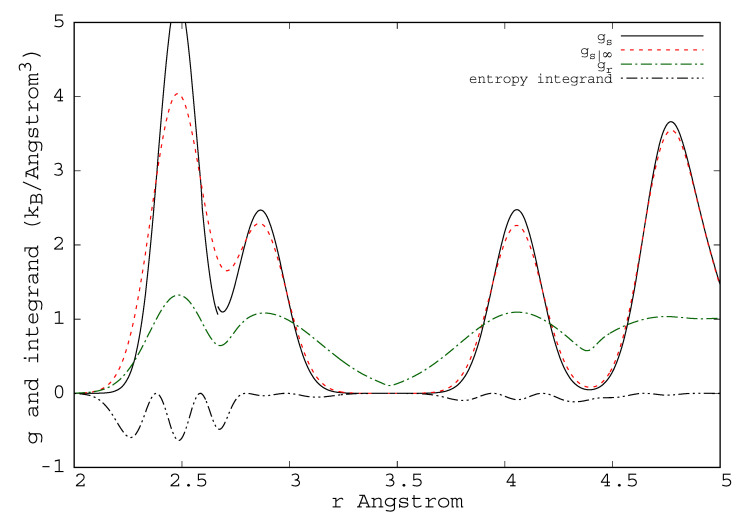
The pair correlation function $g_{s}^{H}\left(|r|\right)$ for BCC iron at 500 K, as simulated with the Johnson potential, is shown as a solid line. The function $g_{s|\infty }^{H}$ that is associated with the reference entropy is shown as a dashed line. It is the spherical average of Gaussians of width, $\lambda_{\infty }$ centered at each of the lattice separation vectors. The ratio, $g^{r}\left(|r|\right)$ is shown as a dash–dot line; it goes to 1 at large separation. The combination $-\frac{4\pi r^{2}}{2}g_{s}^{H}(\ln g^{r}-\left(1-1/g^{r}\right)$ is the integrand that determines the entropy correction with the reference entropy from the spherical treatment of the Kirkwood approximation in the crystal (dash–dot–dot).

For crystalline pair correlation functions our proposal to use the definition of PCF for inhomogeneous densities (Equation (24) gives, when $g^{r}=1$, a “reference” entropy, $s_{K}^{x}\left[1\right]$. This definition extends the Kirkwood approximation of crystals. The reference entropy, $s_{K}^{x}\left[1\right]$, does not account for correlation between atoms on different sites when g≠1. In Figure 5 the reference entropy provides a smooth continuation of the modified Kirkwood entropy of the fluid; it misses the drop in entropy at solidification. Figure 5 also shows several treatments that approximately incorporate correlation between atoms on different sites. These approximations are motivated by the Kirkwood approximation, conditional probabilities, and the Morris and Ho application of harmonic entropy to crystals. Our relabeling scheme leads naturally to entropy reduction being associated with nearest neighbors along a chain, $s_{K}^{x}\left[G_{\lambda_{01}}\left(R_{1},r\right)\right]$. We found that for the Johnson potential the neighbors beyond first neighbors contribute very little to entropy reduction; therefore, in Figure 5 we show (see unfilled triangles) only $s_{K}^{x}\left[g\left(r\right)\right]$, which includes the correlation between all sites along the chain. The unfilled diamonds show $s_{K}^{x}[g_{s}\left(|r|)\right]$, which is based on the spherically averaged *g*; it is very similar in implementation to the Kirkwood approximation in the fluid (see Figure 6). The two evaluations of the $s_{K}^{x}$ functional at g(r) and $g_{s}\left(|r|\right)$, i.e., $s_{K}^{x}\left[g\left(r\right)\right]$ and $s_{K}^{x}[g_{s}\left(|r|)\right]$ give similar values. The similarity of these results is due to a cancellation of effects; the spherical treatment accounts for a higher coordination but with a larger variation. Consider, along the chain each site shares two bonds with its two neighbors specified by three coordinates (three coordinates per site), while the spherical treatment shares bonds with eight neighbors specified by one coordinate (four coordinates per site).

The Morris and Ho approach, applied using the Einstein Hamiltonian as an approximate potential, gives exactly the reference entropy; $s_{I\;h}^{x}=s_{K}^{x}\left[1\right]$. Application to a nearest neighbor elastic rod model (restoring forces in three directions) gives an expression, $s_{\mathrm{II}\;h}^{x}$, for the entropy similar to $s_{K}^{x}\left[G\left(R_{01},r_{1}\right)\right]$ but which gives a slightly higher entropy. Including interactions between neighboring rods has little impact, $s_{\mathrm{II}\;h}^{x}\approx s_{\mathrm{II}\;h-\mathrm{TT}}^{x}$. As $s_{K}^{x}\left[G\left(R_{01},r_{1}\right)\right]\approx s_{K}^{x}\left[g\left(r\right)\right]$ we have represented the difference between the Morris Ho approach and the Kirkwood approach by showing $s_{\mathrm{II}\;h-\mathrm{TT}}^{x}$, filled triangles, and $s_{K}^{x}\left[g\left(r\right)\right]$, unfilled triangles if Figure 5. Both results include entropy reducing correlation between sites; as a result they give an entropy of fusion whereas the reference entropy did not. It is sensible that $s_{\mathrm{II}\;h-\mathrm{TT}}^{x}$ is higher than $s_{K}^{x}\left[g\left(r\right)\right]$ because $s_{\mathrm{II}\;h-\mathrm{TT}}^{x}$ is restricted to be an upper bound. Finally, let us look at inclusion of correlation between nearest neighbors in the Einstein model. The Einstein model performs poorly, it missed the entropy of fusion; however, the correction due to neighbors is large. The correction actually over estimates the entropy of fusion and brings $s_{I\;h-\mathrm{TT}}^{x}$ slightly below the target entropy. In the lower plot of Figure 5 we compare our result to the entropy of a classical density functional theory functional [28] of density and pair potential; the functional was evaluated for the liquid density and the Johnson potential. The entropy related to this functional (Equation (40) of Lutsko and Lam [28]) is:

(64) $$s_{x}^{\mathrm{Lutsko}}\left(T\right)=-\frac{1}{N}\frac{dF_{ex}^{\mathrm{Lutsko}}\left(T,\left[\rho ,v_{\mathrm{Johnson}}\right]\right)}{dT}.$$

The agreement with the target of the various approximations is good considering the simplicity of the functionals. In future work, based on a more diverse set of simulation data, any of the approaches presented here could prove to be the better starting point for accurate representations of the universal excess entropy functional.

## 5. Conclusions

The important message of this work is not the quality or lack of quality of our proposed excess entropy functionals, but rather, the existence of a universal excess entropy functional and the usefulness of an entropy functional approach. We foresee that further research will bring a broadly applicable, accurate functional not only for single component systems but also for multicomponent, molecular, macroscopic particle, and spin systems. More imaginative functional forms that are designed to satisfy a variety of formal restrictions and fitted to vastly greater amounts of simulated data will advance the development of the functionals. Here we have mapped out a few starting points for functional development in order to clarify the entropy functional machinery. We have also put forth physical interpretations relating features of the PCFs and their role in entropy reduction; these relationships are obscured when the entropy is obtained through thermodynamic integration. The pair entropy functional approach could accelerate the study of technologically interesting materials and materials processes. For both first principles (DFT) simulations and simulations with classical many-body interaction (see Appendix D), entropy functional evaluation will make determination of the free energy as routine as the evaluation of the energy.

## Figures and Tables

**Figure A1:**
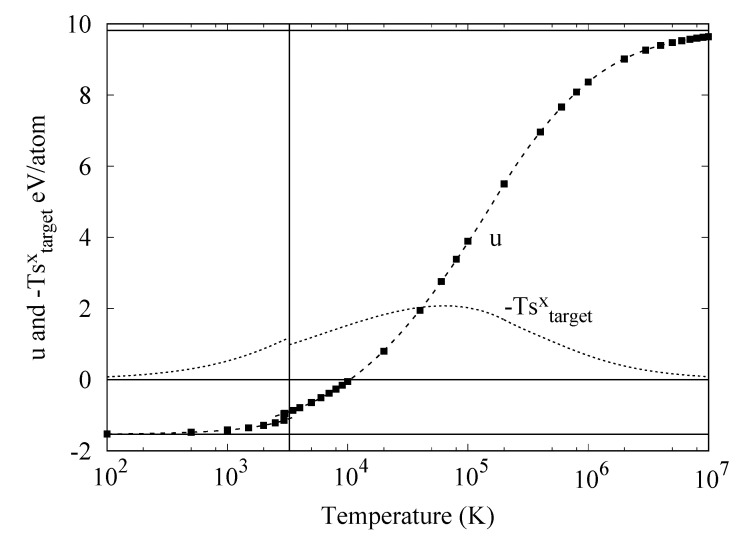
The potential energy, *u*, and the negative of the entropy multiplied by *T*, $-Ts_{target}^{x}$, in (eV/atom) are plotted as a function of *T* for iron as simulated with the Johnson potential. The dashed line is the fit to the simulated potential energy as described in the text. The dotted curve is obtained analytically from the fit to u(T). The symbols are the values of the simulated potential energy at the set of simulation temperatures used for the fit. Horizontal solid lines indicate the exact T=0 potential energy, the zero of energy, and the exact T=∞ potential energy. The vertical line is at $T_{m}$.

## Data Availability

This manuscript has been authored by UT-Battelle, LLC under contract number DE-AC05-00OR22725 with the U.S. Department of Energy. The United States Government retains, and the publisher, by accepting the article for publication, acknowledges that the United States Government retains a non-exclusive, paid-up, irrevocable, world-wide license to publish or reproduce the published form of this manuscript, or allow others to do so, for United States Government purposes. The Department of Energy will provide public access to these results of federally sponsored research in accordance with the DOE Public Access Plan (http://energy.gov/downloads/doe-public-access-plan (accessed on 11 February 2021)).

## Appendix A. Universal Functionals

To establish the position of this work within several approaches based on universal functionals, we give a brief survey of some relevant universal functional theories.

Density function theory (DFT) [61] for electrons addresses the quantum (usually Fermion) many-body problem where the interaction is unchanging; it is the pairwise, Coulomb interaction between electrons (many body interactions are zero). The underlying minimization principle is the Raleigh–Ritz variational principle of the ground state energy (expectation value of the Hamiltonian) with respect to the many-body wave-function. The problematic terms are the interaction energy (including exchange) and the kinetic energy; the straightforward term is the external energy (energy of interaction with the point potential that usually arises from Coulomb interactions with fixed protons). The external energy is easily determined from knowledge of the density, $E_{\mathrm{external}}=\int dr\rho \left(r\right)v^{\left(1\right)}\left(r\right)$. If the point potential is changed, i.e., protons are displaced, the energy changes; this contributes to forces between protons exerted via the electrons, forces that can be used in classical DFT-molecular-dynamics. The sum of electron kinetic and interaction energy is a universal functional of the density; the functional is independent of the point potential generated by the protons; the functional is system independent. The majority of proposed approximations to the universal functional are based on the kinetic and interaction energy for a particularly simple point potential, specifically $v^{\left(1\right)}\left(r\right)=0$. DFT was originally derived based on the uniqueness of the point potential that generates a given density [61]. More recently DFT has been derived from a constrained search procedure [36]. Typical calculations assume zero temperature; however, electron entropy at finite temperature is sometimes addressed [33]; furthermore, DFT-MD is often performed with the classical ions at a finite temperature set by a thermostat. Research in DFT strives to develop more accurate or simpler approximations to the universal functional, evaluate the functional for larger or more complex systems, or apply DFT to systems of physical, chemical, or technological interest. Electron DFT has proved to be a very successful computational approach. The quality of approximate functionals has improved through decades of research. It is instructive to note that a large number of approximate functionals that have been developed to the point of being incorporated into packaged DFT codes, [62]. Based on DFT it can be said that given a ground state density, and no other information, it is possible to determine the kinetic and interaction energy by inserting the density into the universal functional. Furthermore, the variation of the functional with respect to the density gives the external potential, thus determining the ground state energy of the electron system.

Classical density functional theory, CDFT [63,64], addresses the classical many-body problem ( in or near equilibrium) where the interaction between particles is typically different for each system studied and different for each of the types of particles within the system. The underlying minimization principle is of the free energy with respect to variation of the probability density. The most problematic term is the entropy. The kinetic energy is that of the ideal gas. The external energy is given trivially in terms of the density, $E_{\mathrm{external}}=\int dr\rho \left(r\right)v^{\left(1\right)}\left(r\right)$. The interaction energy is given straightforwardly in terms of the correlation functions (just pair correlation for pair interactions). For the interaction energy to be expressed as a functional of the density, an expression that relates the correlation to the density is required. Similar to the situation for electron DFT, this relationship is usually developed for the case when $v^{\left(1\right)}\left(r\right)=0$. Unlike electron DFT, which applies to a fixed interaction, in CDFT the relationship is reestablished through MD or Monte Carlo for the interaction and temperature of interest. Similar to electron DFT, the universality of the functional rests on the proof that, for a given set of fixed interactions, the point potential that generates a particular density is unique. In applications, the external potential is often the chemical potential of involved phases. The density changes through, for example, a solid liquid interface. Much of the recent work on CDFT has been in the context of Phase Field Crystal (PFC). Much of the work in PFC has been on developing an approximate functional that displays stability for relevant phases (liquid, BCC, FCC,...) as a function of temperature, [65]. Research in CDFT covers the same types of issues as encountered in electron DFT. In a special issue of Journal Physics: Condensed Matter, *New developments in Classical Density Functional Theory* [29], applications of CDFT are described along with recent improvements in functionals.

Pair-DFT for electrons has been proposed, [66]. In Pair-DFT the kinetic energy is a universal functional of the spin resolved pair density and the interaction energy is a simple functional of the pair density and pair interaction. Pair-DFT could utilize an effective two electron Schrödinger equation in a similar fashion to the Kohn-Sham approach [67] to DFT which is based on an effective one electron Schrödinger equation. A more pragmatic approach could combine selected results from full pair-DFT with improved relationships between the pair-density and the density to yield improved DFT approximations. In pair-DFT, given a density and the electron-electron pair correlation functions $g_{\alpha ,\beta }\left(r,r^{\prime }\right)$, the electron kinetic energy could be obtained by simply plunging them into the pair-DFT universal functional (with no knowledge of the point potential and pair interaction).

As discussed in this paper, pair-CDFT boils down to obtaining the excess entropy from the density and pair correlation function (no other information required). For clarity we have focused on the homogeneous case, i.e., $v^{\left(1\right)}=0$. Furthermore, the aspects associated with a point potential are already developed in CDFT. CDFT can be recovered from pair-CDFT by relating the pair correlation function to the density; in forming this relationship to recover a CDFT it is permissible to use the pair interaction because, after all, CDFT is for a specific, fixed interaction.

## Appendix B. Stationarity and the Unique Potential

The minimization with respect to *g* codified in Equation (13) implies, subject to continuity restrictions, stationarity of A[g] with respect to *g*. How can stationarity with respect to *g* be utilized? Three situations come to mind:

1. Both v(r) and g(r) are known;
2. Only v(r) is known;
3. Only g(r) is known.

If an accurate and easily evaluated universal $S_{x}\left[g\right]$ has been developed, then for situation (1) the free energy can be obtained by plugging v(r) and g(r) into Equation (20). For example, if a simulation with a known potential has been performed and *g* has been determined from the output; the free energy can be obtained without thermodynamic integration simply by substitution. Furthermore, the value obtained is expected to be reliable because of the stationarity of A[g]. Situation (2) applies when v(r) is known but no simulation or measurement of *g* has been performed; in this case *g* could be found, not from simulation, but from the requirements that it minimize *A* and that *A* be stationary at the true *g*. The free energy could then be obtained by substitution. Situation (3) could arise when only *g* is known, for example, a measured *g*. Stationarity allows the unique potential to be determined from A[g] at any *T*:

(A1) $$0=\frac{\delta A}{\delta g}=\frac{N}{2}v\left(r\right)-T\frac{\delta S_{x}\left[g\right]}{\delta g}.$$

As a simple illustration, consider the dilute limit where $g\approx \exp(-\frac{v(|r|)}{k_{B}T})$ and the excess entropy is approximated by: $S_{x}^{0}=-N/2\int^{R}dr\left(g\ln g-\left(g-1\right)\right)-N$; Equation (A1) is satisfied exactly. In general, if the excess entropy functional expressions were found that were both accurate and had algebraic variations with respect to *g* then the pair potential would be given explicitly in terms of *g*. Therefore, an accurate approximation of the entropy functional eliminates the need for reverse Monte Carlo. Furthermore, according to Equation (A1) once the pair potential is known the potential energy and hence the free energy is specified.

(A2) $$A\left[g\right]=A^{id}+T\left(\int drg\left(r\right)\frac{\delta S_{x}\left[g\right]}{\delta g}-S_{x}\left[g\right]\right)$$

Note that Equation (A2) specifies *A* in terms of *g* but it is not stationary in *g*, essentially, because stationarity was already used to eliminate v(r). If *g* were known from experiment or simulation Equation (A2) would give *A* provided that the interactions were pairwise.

Equations (A1) and (A2) go beyond Henderson’s theorem [23] by specifying the relationship between the entropy and the unique potential and between *A*, $S_{x}$, and *T*. If present, the impact of the many-body interactions would manifest in both the pair (measured or simulated) and the higher order correlations (typically these are not measured and typically these are not calculated from simulations).

## Appendix C. Mapping Atomic Coordinates to Atom List

Arranging atoms along a linear chain that travels from neighbor to neighbor helps clarify our discussion. For a given set of atomic coordinates this is not a unique operation. Furthermore, the atoms move as a simulation progresses. The results derived in this paper are not sensitive to the details of list construction. We do not foresee that list construction will be incorporated as part of simulation, it is a gedanken-construction. Our proposal is: (1) for the set of atomic positions perform a steepest descent to the inherent structure; (2) find the shortest chain or chains that links all sites with nearest neighbor links; (3) index atomic positions according to the position in the chain of the inherent structure site that it descended to in the steepest descent process; (4) if there are multiple chains of the same length calculate the entropy employing each chain and average. For a crystal simulation there would be multiple chains but each would give the same result so averaging is not needed. In most cases the differences between chains would go to zero in the thermodynamic limit so no averaging would be needed. However, there could be cases, e.g., defects, where special care would be needed to represent and average over bonds local to the defect.

## Appendix D. Inclusion of Many-Body Effects

In order to obtain many body corrections to *A* from the Gibbs-Bugoliubov inequality the higher order correlation and the, presumably small, many body interactions would have to be known. Unfortunately neither the interactions nor the higher order correlations associated with an experimental *g* are ever exactly known. However, the excess-entropy functional does not suffer the same limitation because it is defined in Equation (18) by a constrained search. The linear change in $S_{x}$ due to the change in *g* under the influence of many-body interactions is included because it is the modified *g* that is known. In the presence of many-body interactions the entropy functional, $S_{x}^{pair}$, is modified by constraints on *P*, becoming $S_{x}^{manybody}\left[g,...g^{n}\right]$. The *P* that maximizes Equation (18) will differ from the constrained true many-body probability density by an amount proportional to the many-body interactions but which will lower $S_{x}$ by an amount quadratic in the many-body interactions $(S_{x}\left[g\right]=\max_{g^{\left(n>2\right)}}S_{x}\left[g,g^{\left(3\right)},g^{\left(4\right)},\cdots \right]$ $=S_{x}\left[g^{\left(2\right)},g_{max}^{\left(3\right)},g_{max}^{\left(4\right)},\cdots \right]\to S_{x}^{manybody}$ $=S_{x}^{pair}\left[g\right]-O\left({\left(v^{\left(n\right)}\right)}^{2}\right)$. The entropy functional of Equation (18) provides a stationary upper bound to the entropy with respect to many-body interactions. The same argument applies to g(r) from first principles MD; the entropy could be well reproduced by Equation (18) because the effect of higher order interaction is second order. The internal energy (total energy) from the first principles MD can be added to $-TS^{id}\left(T\right)-TS_{x}\left[g\right]$ to obtain a first principles *A* that is accurate to second order in the difference between the first principles interactions and an, unspecified, effective pair potential. Knowledge of the functional, $S_{x}\left[g\right]$, provides a general method for obtaining the free energy from first principles without thermodynamic integration.

## Appendix E. Simulation Results and Target Entropy from Johnson Potential

Our procedure for calculating the target entropy started with simulations at 40 temperatures between $10^{0}K$ and $10^{7}K$. The simulation cell contained 16,000 atoms with periodic boundary conditions in a cube shaped supercell with volume $8000a^{3}$, where a=2.86997Å is the lattice constant. The Nose-Hoover thermostat was used. At each temperature the system was equilibrated until energy convergence was reached. Data collection runs of 1ns with configurations saved every 0.01ps provided data for calculation of the pair correlation functions. The configurations were post processed to produce the $g_{s}\left(|r|\right)$ and $\bar{g}\left(r\right)$ at each temperature. They were inserted into the excess entropy functionals to produce Figure 5. A series of two-phase coexistence [68,69] simulations performed over temperatures near melting were used to determine $T_{m}$ by the stationary-phase-boundary-method; $T_{m}=3278K$. The simulated potential energies, u(T), were fitted to three separate forms in three temperature ranges: (1) for temperatures below $T_{m}$, u(T) was fitted to a cubic polynomial in *T*, (2) for $T_{m}<T<T_{t}=200000K$, u(T) was fitted to a quartic polynomial in ln(T), and (3) for $T>T_{t}$, u(T) was fitted to a fifth order polynomial in $\frac{1}{T}$. By construction the analytic expressions were restricted to give the exact values for u(T=0), $\frac{d\;u(T)}{d\;T}{|}_{T=0}$ and u(T=∞) and to join smoothly at $T_{t}$ [70].

(A3) $$u\left(T\right)=\left\{\begin{array}{cc} u_{0}+\frac{3}{2}k_{B}T+u_{2}T^{2}+u_{3}T^{3}\; & T<T_{m};\\ u_{t}-\left(a_{l}*\left(\ln\left(T\right)-l_{t}\right)+b_{l}\left(\ln{\left(T\right)}^{2}-l_{t}^{2}\right)+c_{l}\left(\ln{\left(T\right)}^{3}-l_{t}^{3}\right)+d_{l}\left(\ln{\left(T\right)}^{4}-l_{t}^{4}\right)\right)\; & T_{m}<T<T_{t};\\ a-T_{0}/T+bT_{0}^{2}/T^{2}+cT_{0}^{3}/T^{3}+dT_{0}^{4}/T^{4}+eT_{0}^{5}/T^{5}\; & T\geq T_{t}. \end{array}\right.$$

The entropy was obtained analytically from the parameterized form for u(T). Analytic integration from T=∞ to *T* gave the entropy above $T_{m}$. At $T_{m}$ the entropy was reduced by the entropy of fusion, $s_{f}=\frac{u{\left(T_{m}\right)}_{liquid}-u{\left(T_{m}\right)}_{crystal}}{T_{m}}$. Analytic integration was then continued from $T_{m}$ to *T* to obtain the crystal entropy down to 1K. Figure A1 shows the simulated u(T) and $-Ts_{target}^{x}\left(T\right)$ where $s_{target}^{x}\left(T\right)$ is the target excess entropy. This target entropy was used to fit $q_{0},q_{1}$ and $q_{2}$ by straightforward linear regression [51].

## Appendix F. Classical Debye Approximation of *ϵ*

The ratio, *R*, of the variance of the separation vector of a pair of nearest neighbors atoms to the variance of displacement of an atom about its average position is [52]:

(A4) $$\begin{array}{ccc} R_{1nn} & = & \frac{\sum_{i}<{\left|u_{i}^{1}-u_{i}^{0}\right|}^{2}>}{\sum_{i}<{\left|u_{i}^{0}\right|}^{2}>} \end{array}$$

(A5) $$\begin{array}{ccc} & = & \frac{\int dq|\exp\left(iq\cdot n_{1}\right){-1|}^{2}\frac{\left({\bar{n}}_{q}+\frac{1}{2}\right)}{\omega (q)}}{\int dq\frac{\left({\bar{n}}_{q}+\frac{1}{2}\right)}{\omega (q)}} \end{array}$$

In the classical limit:

(A6) $${\bar{n}}_{q}+\frac{1}{2}=\frac{3T}{2\omega (q)}.$$

For isotropic acoustic phonons:

(A7) $$\omega \left(q\right)=v_{s}q$$

We can evaluate the ratio in (A5) by approximating the Brillouin zone by a sphere of radius $q_{\max}=\frac{{\left(12\pi^{2}\right)}^{\frac{1}{3}}}{a}$, where *a* is the BCC lattice constant:

(A8) $$\begin{array}{ccc} R_{1nn} & = & 2-2\frac{\int_{0}^{q_{max}}dq\frac{\sin(qn_{1})}{qn_{1}}}{q_{max}} \end{array}$$

(A9) $$\begin{array}{ccc} & = & 2-2\frac{\mathrm{Si}(q_{max}n_{1})}{q_{max}n_{1}} \end{array}$$

(A10) $$\begin{array}{ccc} & = & 1.197 \end{array}$$

The ratio for the second nearest neighbor has the value, $R_{2nn}=1.362$ and for very distant neighbors, $R_{\infty }=2$

(A11) $$\begin{array}{ccc} |\epsilon_{I}| & = & |1-\frac{R_{1nn}}{2}|=0.40 \end{array}$$

(A12) $$\begin{array}{ccc} |\epsilon_{II}| & = & |1-\frac{R_{2nn}}{2R_{1nn}}|=0.43 \end{array}$$
